# A novel calix[4]pyrrole derivative as a potential anticancer agent that forms genotoxic adducts with DNA

**DOI:** 10.1038/s41598-018-29314-9

**Published:** 2018-07-23

**Authors:** Marta Geretto, Marco Ponassi, Martina Casale, Alessandra Pulliero, Grazia Cafeo, Ferdinando Malagreca, Aldo Profumo, Enrica Balza, Rakhmetkazhi Bersimbaev, Franz Heinrich Kohnke, Camillo Rosano, Alberto Izzotti

**Affiliations:** 1 Department of Health Sciences, University of Genova, Genova, Italy; 2 IRCCS Ospedale Policlinico San Martino, Genova, Italy; 3 Department CHIBIOFARAM, University of Messina, Messina, Italy; 4 Department of General Biology and Genomics, Institute of Cell Biology and Biotechnology, L.N. Gumyliov Eurasian National University, Astana, Kazakhstan

## Abstract

*meso*-(*p*-acetamidophenyl)-calix[4]pyrrole **3** was found to exhibit remarkable cytotoxicity towards A549 cancer cells. A comparative study including the isomer of **3** *meso*-(*m*-acetamidophenyl)-calix[4]pyrrole **5**, as well as molecules containing ‘fragments’ of these structures, demonstrated that both the calix[4]pyrrole and the acetamidophenyl units are essential for high cytotoxicity. Although calix[4]pyrroles and other anion-complexing ionophores have recently been reported to induce apoptosis by perturbing cellular chloride concentrations, in our study an alternative mechanism has emerged, as proven by the isolation of covalent DNA adducts revealed by the ^32^P postlabelling technique. Preliminary pharmacokinetic studies indicate that **3** is able to cross the Blood-Brain-Barrier, therefore being a potential drug that could kill primary and brain metastatic cancer cells simultaneously.

## Introduction

**Figure 1:**
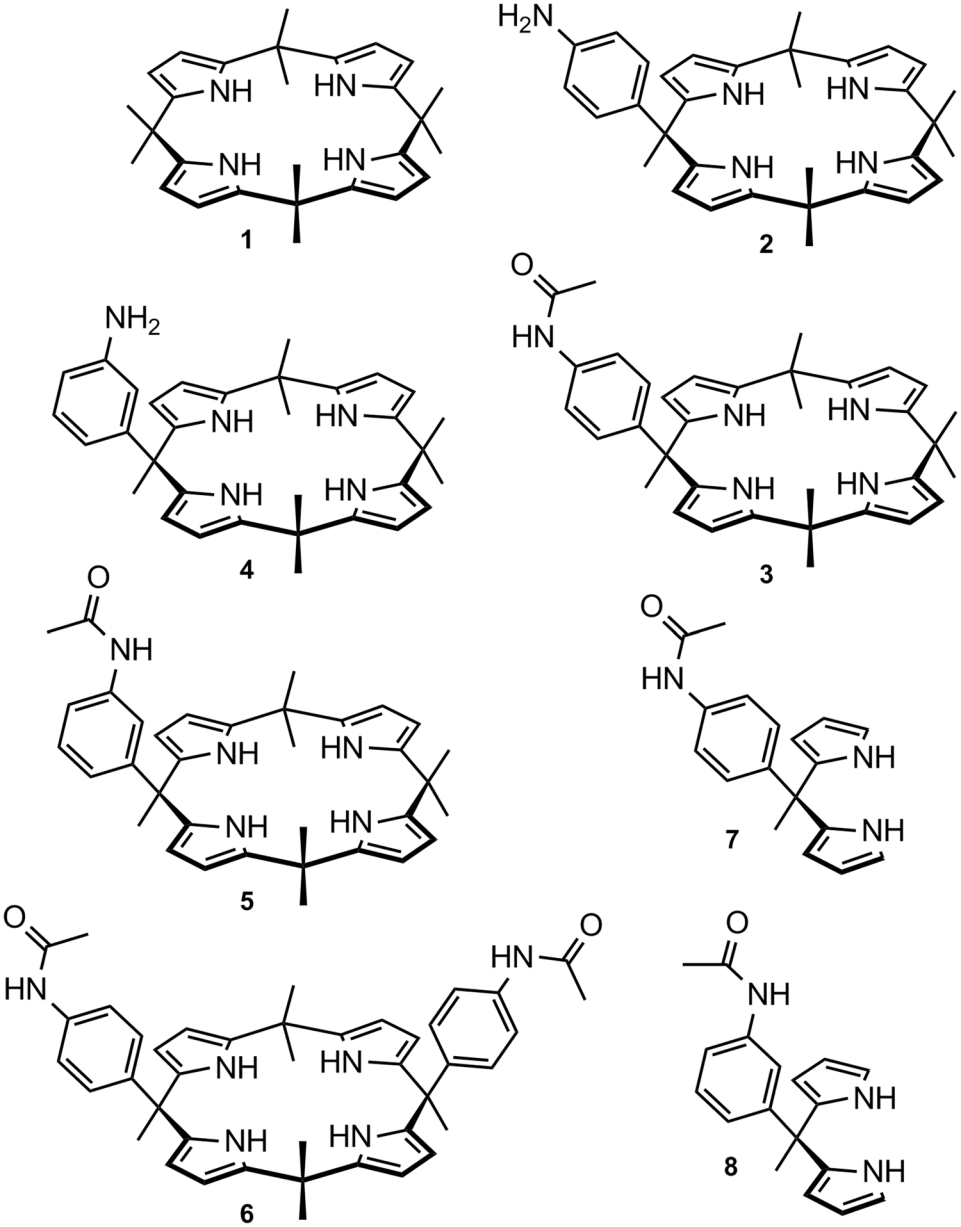
Structural formulae for *meso*-octamethyl-calix[4]pyrrole **1** and for the related compounds tested in this study.

Calix[n]pyrroles are macrocyclic compounds made up of pyrrole units linked at their 2,5-positions by quaternary carbon atoms^1^. *meso*-Octamethyl-calix[4]pyrrole **1** (Fig. 1) has been known for over a century^2^, but interest in this compound (and its congeners) rapidly developed only following the discovery of its ability to form complexes with anions^3^ and neutral molecules^4^ that can accept hydrogen bonds from the pyrrole NH units. Since these seminal papers, a vast number of calixpyrrole derivatives have been synthesised and investigated as selective ligands for different anions^1,5^, for sensing applications^6^, in the assembly of novel materials^7,8^ and devices^9,10^. When developing our early work on heterocyclophanes^11^ and calixarenes^12^ that can bind biologically relevant species, we reported the ability of *meso*-*p*-aminophenylcalix[4]pyrrole **2** to form a cytotoxic *trans*-Pt(II) complex in which the calix unit appears to assist the delivery of the toxic metal to DNA via the preliminary binding of the phosphate residues^13^. In this work, we propose a mechanism by which ‘free’ non-Pt(II) coordinated calixpyrrole **2** is released within the cell when the metal leaves the aminophenyl coordination site of **2** to form new bonds with nitrogen atoms of the nucleobases. Since tests conducted with ‘free’ **2** did not reveal any significant cytotoxicity when this was used at concentrations analogous to that of its Pt(II) complex, it was evident that **2** acted merely as a vector capable of delivering the toxic metal to DNA. To the best of our knowledge, this is the first report on the use of a calixpyrrole derivative for potential biomedical applications as a drug-delivery system.

The crucial role of ion channels for cell function has been widely recognised^14^, and targeting the ion transport mechanism is known to provide a means of affecting cell viability^15^. Hence, as lipophilic anion-complexing agents capable of transporting anions across lipid membranes, calixpyrroles can be considered potentially cytotoxic. They can be viewed as the anion-binding counterparts of the well-known cation-binding ionophoric antibiotics valinomycin^16^ and nonactin^17^. Studies conducted by J. L. Sessler, P. A. Gale, I. Shin and collaborators have demonstrated that some pyridine diamide strapped calixpyroles can affect chloride transport coupled to sodium cation transport in cells^18^. This leads to increased chloride and sodium concentrations within the cell and ultimately to cell death by apoptosis. This work was later extended to other chloride complexing agents beside calixpyrroles^19^. It was found that a delicate balance has to be achieved between binding strength and lipophilic character for a chloride-binding receptor to achieve effective transport and exhibit cytotoxic properties^19^. In these studies^18^, calixpyrrole **1** was shown (Fig. 1) not to exhibit significant cytotoxicity, therefore we were not surprised that calixpyrole **2** was also found to be non cytotoxic^13^. However, it is reasonable to assume chloride not to be the only anion that can be targeted by using ionophores to achieve a cytotoxic effect. For this reason, we recently reported the synthesised calixpyrrole-based receptors containing two or three calixpyrrole units and evaluated their ability to bind bis-carboxylates that are relevant to cancer physiology^20,21^, planning to investigate their biological activities in future studies. Moreover, we previously reported that the *meso*-octamethyl-calix[4]pyrrole **1** acts as antagonist of the G-protein coupled receptor 30 (GPR30) in different model systems, such as breast tumour cells and cancer-associated fibroblasts^22^. The latter work clearly indicated that calixpyrroles have the potential to elicit a biological response by a mechanism that is not linked to their properties as ionophores, but as ligands of the estrogenic receptor GPR30. We therefore selected compounds **3** and **5** shown in Fig. 1 to expand our study on the biological activity of calix[4]pyrrole derivatives because they can be viewed as substructures of the bis- and tris- aromatic amide-linked calixpyrroles reported by us previously^20^, and they clearly comprise structure **1**.

Here we report on the cytotoxic properties of **3** and **5** against several different cancer cell lines and a limited pharmacokinetic (organotropism) evaluation of compound **3** in mice. The pharmacokinetic study was limited to **3** because this was found to be the most active of the tested compounds against the highly aggressive A549 lung cancer cells.

We were not expecting strong evidence for the formation of covalent DNA adducts to emerge during this study. This unexpected result suggests that specific calix[4]pyrrole derivatives could be used as novel genotoxic drugs inducing apoptosis by a mechanism that is not linked to their activity as ionophores.

## Results

**Figure 2:**
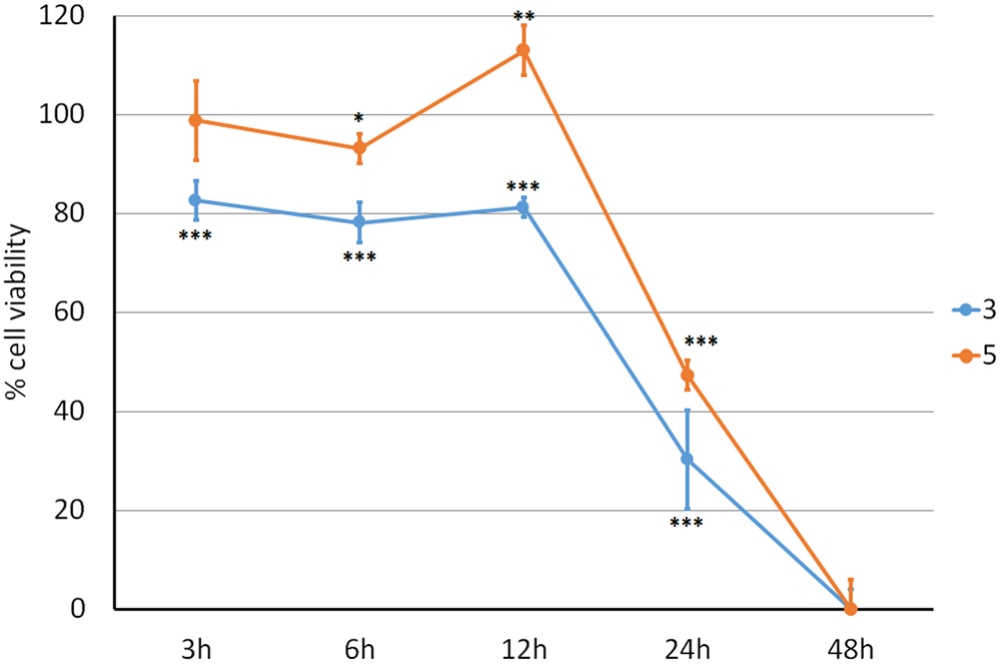
Effects of compounds **3** and **5** on cell viability (crystal violet). Adenocarcinoma A549 lung cancer cell line treated with 5 μM of calix **3** (blue line) and **5** (orange line) shows a time-dependent decreased viability compared to control (100% viability) ***p < 0.001; **p < 0.01; *p < 0.05.

A timing-course experiment performed using **3** or **5** against the A549 lung cancer cell line (Fig. 2) showed that upon treatment with either compound, a high mortality rate (measured by the crystal violet method) occurred at 24 h, while at 3, 6 and 12 h no significant cytotoxic effect could be detected. Cell mortality after 48 hours was 100%.

To elucidate which structural features present in **3** and **5** were necessary for the observed activity, we virtually fragmented these molecules to test their structural components as separate units. This led us to select, synthesise and test the small library of compounds (**2**, **4**, **6**, **7**, **8**) shown in Fig. 1. Although we had already tested **2** in previous studies, this compound was included in the screening for validation purposes. Calixpyrrole **6** (Fig. 1) was included to assess whether the presence of two *p*-acetanilide units would produce enhanced cytotoxicity compared to **3** (which contains only one such unit). Calixpyrrole **6** was obtained by acetylation (acetyl chloride, K_2_CO_3_ in DCM) of the *p*-aminophenyl analogue which has been reported previously^23^. Dipyrromethanes **7** and **8** were prepared by N-acetylation (acetyl chloride, K_2_CO_3_ in DCM) of the previously reported 4-aminophenyl^13^ and 3-aminophenyl^23^ analogues.

After preliminary tests on **3** and **5** using the crystal violet method, we tested the cytotoxic properties of the different calixpyrroles **2**, **3**, **4**, **5, 6**, and of compounds **7** and **8** using the MTT method. Table 1 summarizes the concentrations needed to induce a 50% decrease of cellular viability at the indicated times for the tested compounds against lung, breast, ovary and astrocytoma cancer cells *in vitro*.

**Table 1:** Estimated concentration (d, μM) of compounds necessary to induce a 50% decrease in cellular viability. N.T.: not tested.

Entry	Cell Types	Compounds (see Fig. 1)							Evaluation time in hours
		3	2	5	4	6	7	8	
1	H727 LESS AGGRESSIVE LUNG	d < 5	N.T.	d < 5	N.T.	N.T.	N.T.	N.T.	24.0
2	A549 HIGHLY AGGRESSIVE LUNG	d < 5	50 < d < 100	d < 5	50 < d < 100	d > 100	d > 100	d > 100	24.0
3	MCF-7 BREAST ER+	20 < d < 30	100	10 < d < 20	d > 100	N.T.	d > 100	d > 100	48.0
4	MDA-MB-231 BREAST TRIPLE NEGATIVE	10 < d < 20	50 < d < 100	10 < d < 20	d > 100	N.T.	N.T.	N.T.	48.0
5	SKOV3 OVARIAN	20 < d < 30	50 < d < 100	5 < d < 10	50 < d < 100	N.T.	d > 100	d > 100	48.0
6	U87MG GLIOMA ASTROCYTOMA	50 < d < 100	N.T.	50 < d < 100	N.T.	N.T.	N.T.	N.T.	24.0

**Figure 3:**
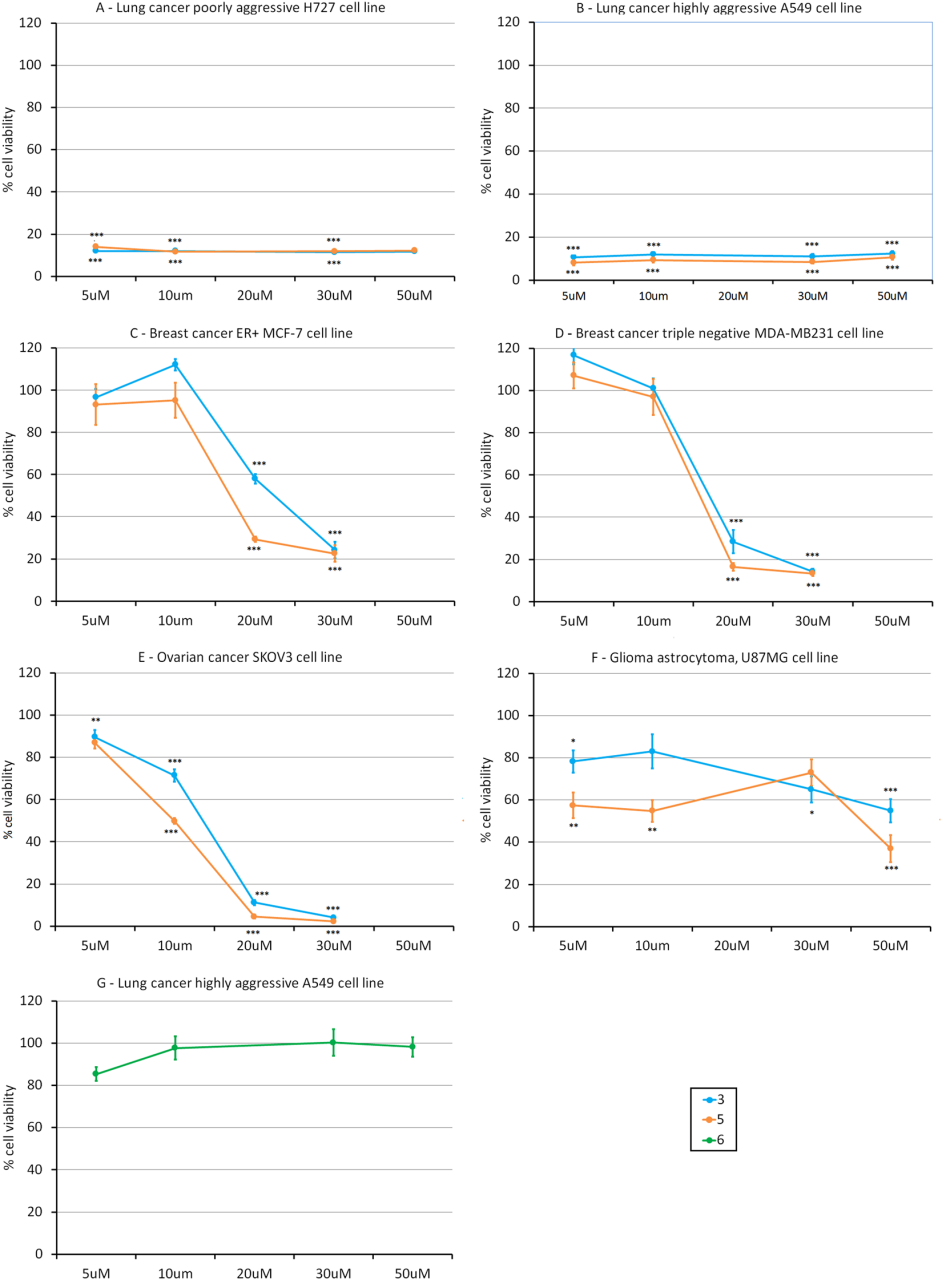
MTT assays on cancer cell lines treated with compounds **3, 5** and **6** at different concentrations (5, 10, 20, 30, 50 μM), for 24 h (Panels A,B and F,G) or 48 h (Panels C,D). ***p < 0.001; **p < 0.01; *p < 0.05. Control corresponds to 100%. Panel A: Lung cancer poorly aggressive H727 cells; Panel B: Lung cancer highly aggressive, A549 cell line; Panel C: Breast cancer ER positive, MCF-7 cell line; Panel D: Breast cancer triple negative, MDA-MB-231 cell line; Panel E: Ovarian cancer, SKOV3 cell line; Panel F: Glioma astrocytoma, U87MG cell line; Panel G: A549 cells treated with compound **6**.

MTT assays performed on the differentiated H727 lung cancer cell line showed compounds **3** and **5** to be effective in decreasing cell viability at the lowest dose (less than 5 μM) with no further significant differences in their behaviour detected compared with the other doses tested (Fig. 3A). Calixpyrrole derivatives were only slightly more effective in decreasing viability of poorly differentiated A549 cells than those of more differentiated H727 cells (Fig. 3A,B, and Table 1 entries 1 and 2 respectively). MTT tests were also performed on U87MG (Astrocytoma), MCF-7, MDA-MB-231 (Breast) and SKOV-3 (Ovarian) cancer cell lines. When used on U87MG astrocytoma cells (Fig. 3F, Table 1 entry 6), compounds **3** and **5** were almost non-effective. Against breast cancer cells MCF-7 and MDA-MB-231 (Fig. 3C,D, and Table 1 entries 3 and 4 respectively), **3** and **5** showed their highest efficacy after 48 h at a dose between 10μM and 30 μM, **5** being slightly more effective than **3**. This activity seems independent of the estrogenic state of the cell line tested (MCF7 is ER + while MDA-MB-231 is triple negative) and it is very limited at doses between 10 μM and 5 μM. However, **3** and **5** were notably more effective against SKOV3 ovarian cancer cell lines (after 48 h of exposure, Fig. 3E, Table 1 entry 5) than against MCF7 and MDA-MB-231. Compound **5** in particular seems to be more efficient than **3** towards this tumour cell line, with an estimated viability of 50% on exposure to a 10 μM dose of compound.

A cellular viability test was then performed exposing A549 lung cancer cell lines to **6**, since this cell type appeared the most affected by **3**. After 24 h, we detected no significant effect even at the higher dose (Fig. 3G, Table 1 entry 2). The dipyrromethane derivatives **7** and **8** were inactive against A549 cancer cells (Table 1 entry 2), these data confirming that both the calix[4]pyrrole moiety and one *meso* acetanilide substituent are necessary components for the cytotoxic properties of **3** and **5**.

**Figure 4:**
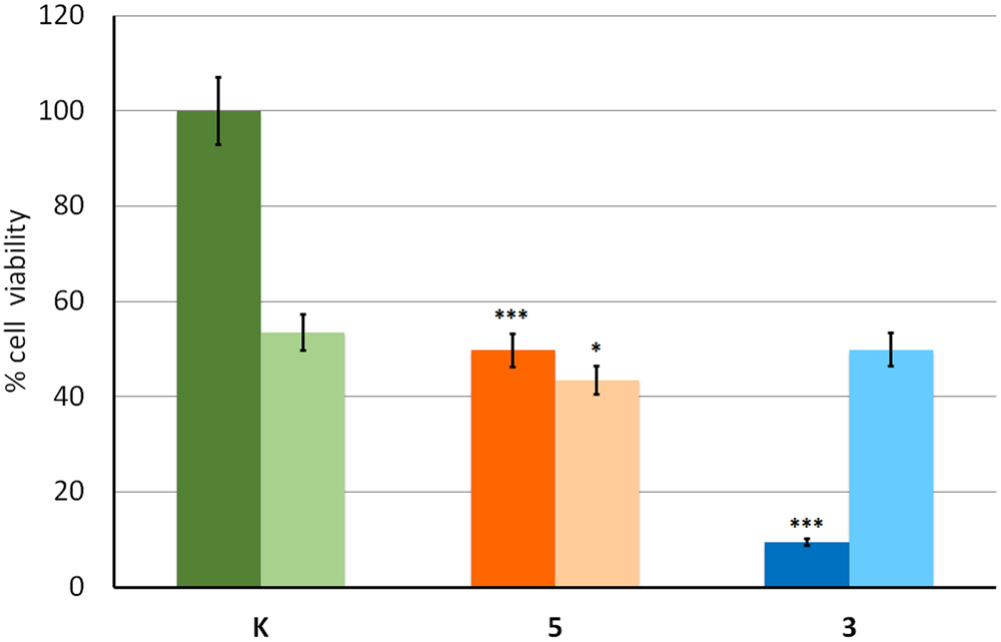
MTT assay on A549 lung cancer cell line using 5μM of the indicated compound in the absence (dark colour) and in the presence (light colour) of the S12 liver fraction (12 h exposure). ***p < 0.001; **p < 0.01; *p < 0.05. K indicates the control.

To shed light on the pharmacodynamics of calix derivatives, we examined the influence of the S12 liver fraction^24^ on drug efficacy. S12 remarkably decreases the effectiveness of calix **3** in killing lung cancer cells (light blue column) as compared to the cytotoxic activity displayed in the absence of S12 (Fig. 4 dark blue column). Thus, **3** undergoes a phase II detoxification reaction in the liver resulting in drug catabolism, detoxification and decreased activity. Accordingly, these molecules, *in vivo*, cannot be administered by the oral route but only by the parenteral route using subcutaneous injection. Intravenous administration should be avoided due to the high lipophilicity of these compounds that could lead to embolism.

**Figure 5:**
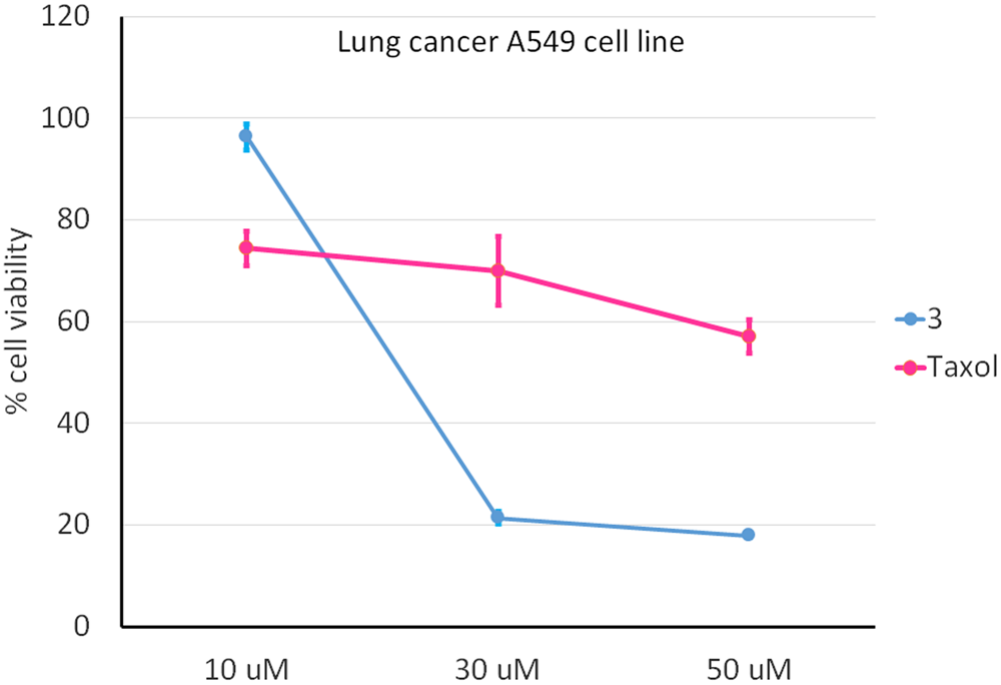
MTT assay on A549 lung cancer cell line treated for 12 h with **3** (blue line) or Taxol (violet line). Control corresponds to 100%, p < 0.001.

The efficacy of **3** against A549 lung cancer cells was compared with that of Taxol. Cells were exposed to increasing concentrations of **3** and Taxol (10, 30, 50 μM, both dispensed with the same solvent, see method section) for 12 h because this is the time-span normally employed to test highly cytotoxic drugs used in chemotherapy such as Taxol. In fact, after longer times the residual viability was too low to allow significant comparisons. At concentrations higher than 20 μM (see the intersection between the two plots in Fig. 5), compound **3** appears to be better at killing cancer cells than Taxol.

**Figure 6:**
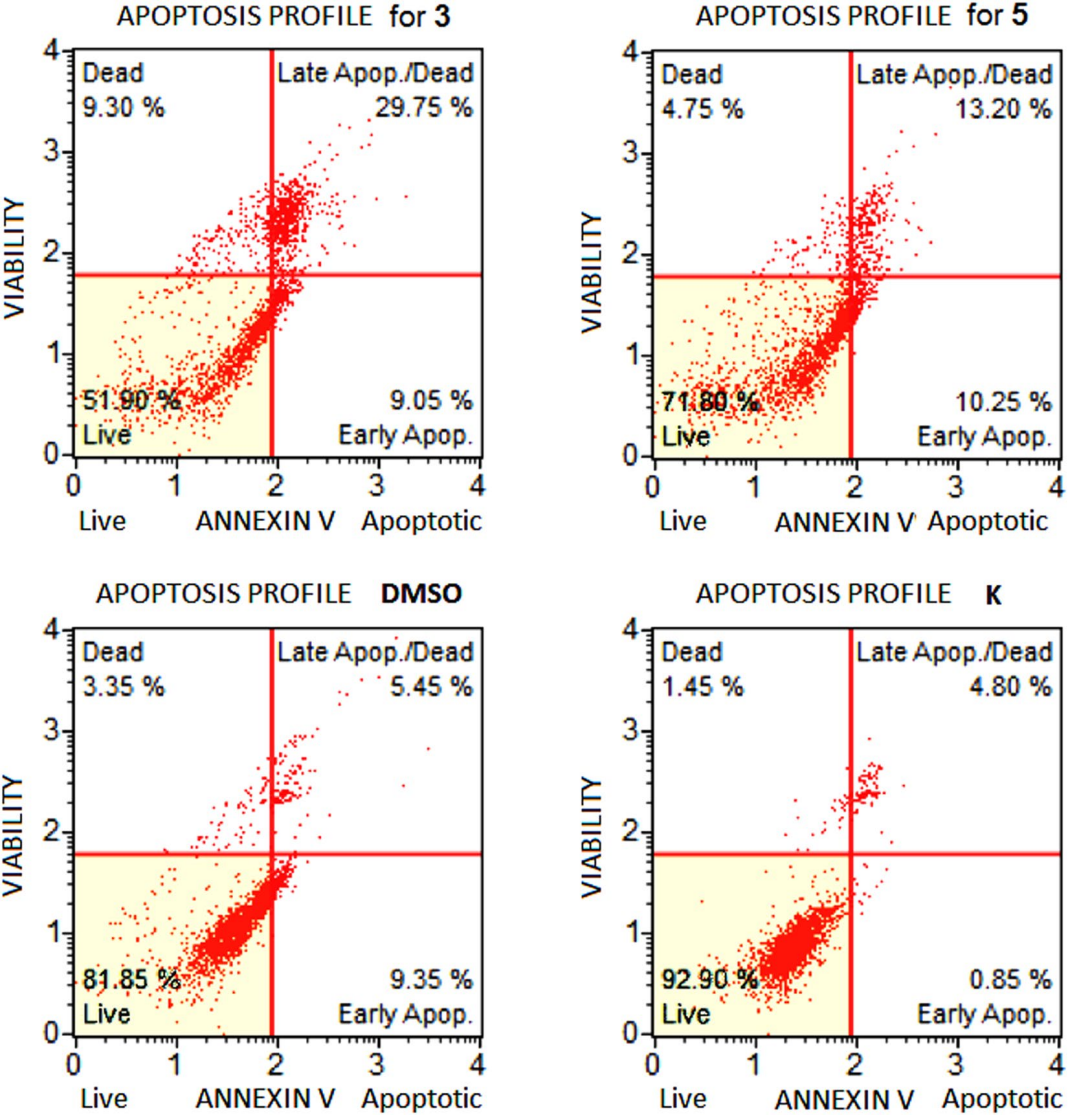
Apoptosis profile (Muse™ Annexin V & Dead Cell Assay) for A549 cells treated with **3** or **5** (5 μM), or DMSO alone (the same amount used to dissolve **3** or **5**: 0.1% of DMSO) and untreated cells (K). Profiles were determined 24 h after treatment in all cases. Each plot has 4 quadrant markers, reflecting the different cellular states: the upper left quadrant contains dead cells (necrosis), the upper right has late apoptotic/dead cells (cells that are positive both for Annexin V and for cell death marker 7-AAD, 7-Aminoactinomycin D), the lower left contains live cells and the lower right early apoptotic cells (cells that are positive only for Annexin V).

Following the evaluation of the cytotoxic properties of compounds **3** and **5** against the above indicated cancer cell lines, we explored the mechanism of action by which cell death occurred. This work was limited to A549 cells, as this cell line was found to be the most affected by these calixpyrrole derivatives. Flow cytometric analysis indicated apoptosis to be the main mechanism. Indeed, A549 cells treated with compound **3** showed 38.80% of total apoptosis (9.05% early apoptosis and 29.75% late apoptosis) compared to 9.30% of necrosis. In the same cell line, compound **5** determined 23.45% of total apoptosis (10.25% early apoptosis and 13.20% late apoptosis) while necrosis accounted for 4.75% (Fig. 6).

**Figure 7:**
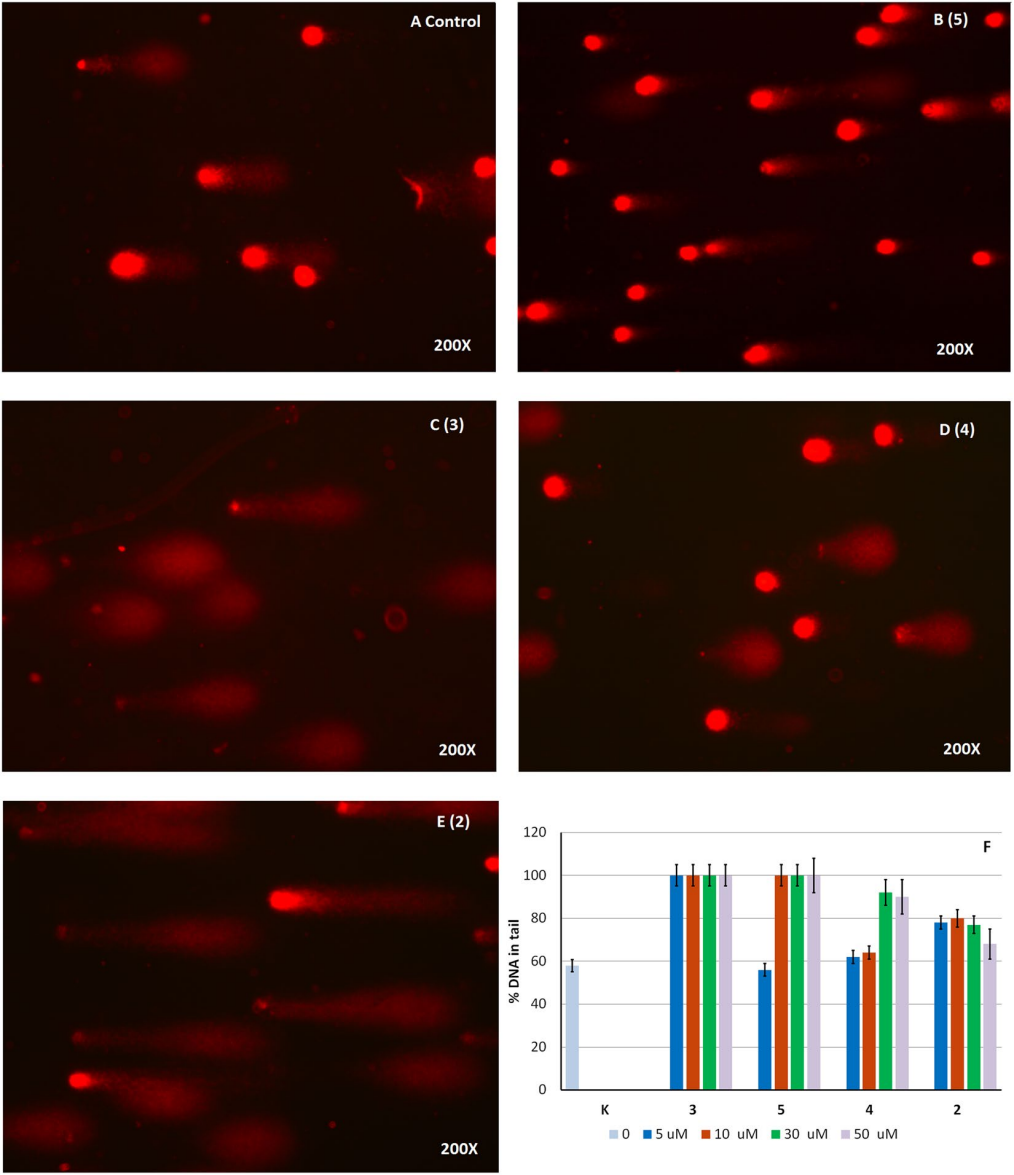
COMET assays on A549 cell line. The assay evaluates DNA fragmentation by quantifying % DNA in tails formed from A549 cell nuclei after treatment with the different calixpyrroles (5–50 μM dose, 24 h in all cases), followed by alkali treatment and single-cell electrophoresis. Microphotographs show the appearance of DNA distribution in ‘comet tails’ following a 5 μM dose treatment: (**A**) sham-treated control; (**B**–**E**) for **5**, **3**, **4**, and **2** respectively. Panel F: % DNA in tail in A549 cells when the test was conducted using the indicated concentrations of compounds **2–5**.

COMET assay (Fig. 7) shows DNA fragmentation in cells treated with compound **3**, which was able to induce massive DNA damage at each concentration tested. However, a higher concentration was required to achieve a similar effect with **5**. Compounds **2** and **4** were significantly less effective than **3** and **5** at all concentrations tested.

**Figure 8:**
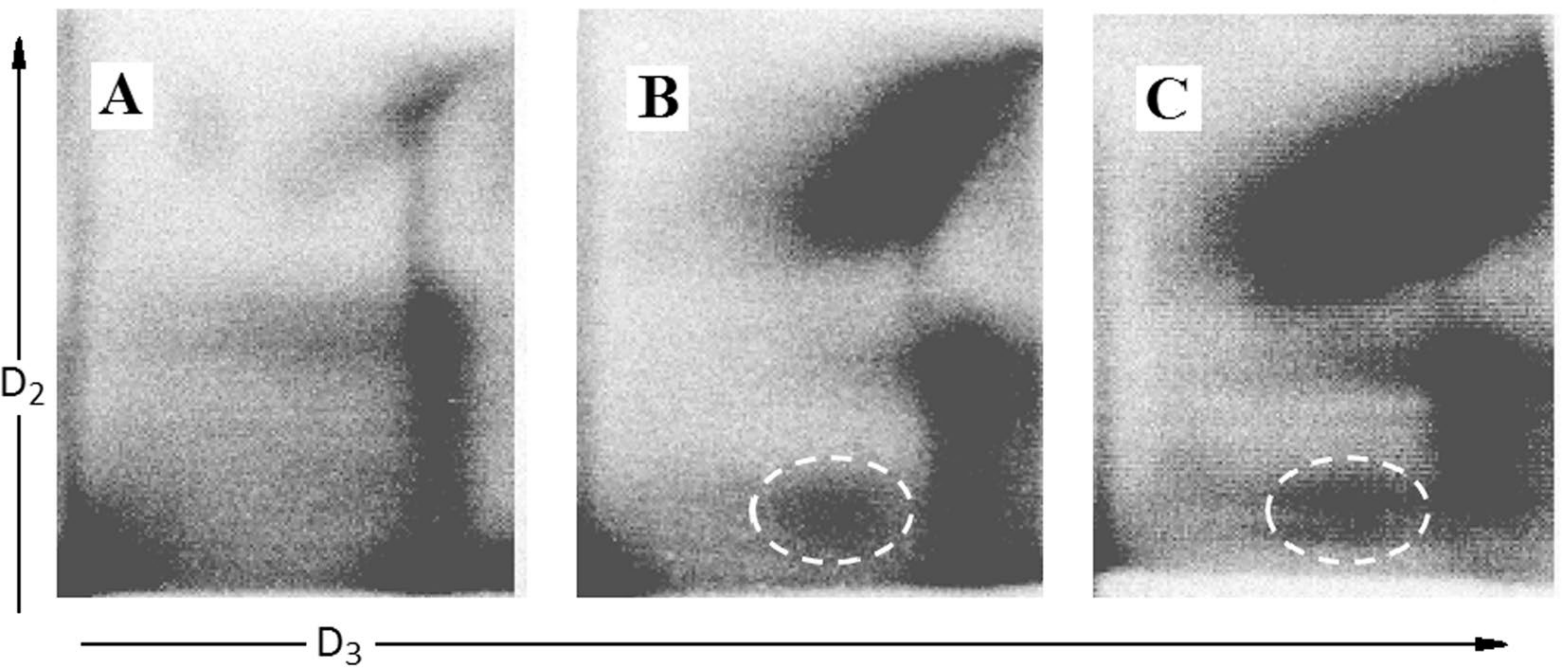
Detection of DNA adduct in A549 cells: sham-treated (panel A); treated with **3** (panel B), or with **5** (panel C). Adducts were labelled by ^32^P and purified by multi-directional thin layer chromatography whose origin is located in the bottom left corners (OR). Adducts (white dashed line circles) are identified as radioactive (black) spots by electronic autoradiography performed by phosphoro-imager. D_2_, direction of second chromatographic elution (urea buffer pH 3.5); D_3_, direction of third chromatography elution (urea buffer pH = 8.5); D_1_ and D_4_ elutions (not reported) with washing buffers (sodium phosphate). D_1_ elution direction was opposite to D_2_; D_4_ elution was the same as D_3_. The black areas on the right correspond to the final edge of the chromatogram.

The ability of calixpyrrole derivatives **3** and **5** to bind DNA was determined by analysing the formation of lipophilic DNA adducts in treated cells by the ^32^P postlabelling technique^25^ (Fig. 8). The DNA of treated cells was depolymerised into single nucleotides, adducts were extracted by washing with water-saturated butanol and labelled by ^32^P using AT-gamma-^32^P as donors and T4 nucleotide kinase. Adducts were resolved by multi-directional thin layer chromatography on polyethylenimine-coated cellulose sheets using sodium phosphate and urea buffers and identified by electronic autoradiography as previously reported^25–27^.

The comparative analysis between controls and calixpyrrole-treated cells revealed the formation of specific DNA adducts only in treated cells, as detected by thin layer chromatography and autoradiography (highlighted by dashed line circles in Fig. 8). These are characterized by the low chromatographic mobility that can be predicted for lipophilic/bulky covalent adducts of **3** or **5** and/or of their metabolites to DNA. These adducts were enriched by butanol extraction of the enzymatically digested DNA, thus being identifiable as highly lipophilic adducts, a characteristic that would arise from the presence of the calixpyrrole component. The adduct amounts were 4.2 ± 0.51 and 4.5 ± 0.28 adducts/10^8^ normal nucleotides for compounds **3** and **5**, respectively (mean ± SD of 3 independent replicated experiments).

### MicroRNA Expression

**Figure 9:**
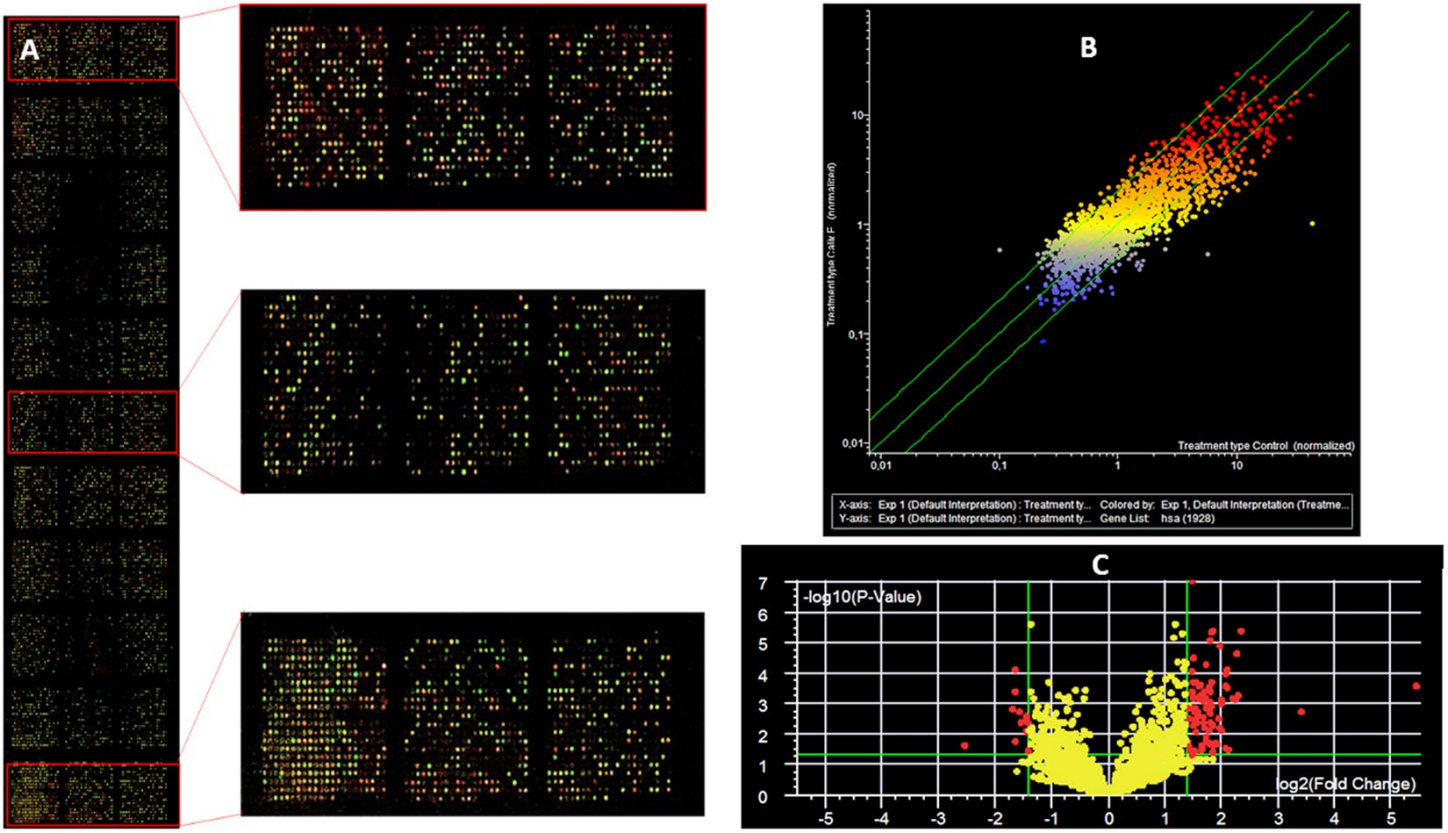
microRNA expression as evaluated by microarray analysis in A549 cells either sham-treated or treated with **3**. Panel A: hybridised microarray showing the fluorescent miRNA captured onto immobilised probes; the call-response rate (i.e. the number of fluorescent spots) is high (>90%) indicating a good quality analysis. Panel B: scatter plot analysis comparing the level of expression of each one of the 1,350 analyzed miRNA (dots) in sham (horizontal axis) versus **3**-treated cells (vertical axis). Dots falling outside the 1.5-fold variation interval (diagonal green lines) are either upregulated (upper left area) or downregulated (lower right area) by **3**. Dot colours indicate the level of miRNA expression (blue low, yellow intermediate, red high). Panel C: Volcano plot analysis of miRNA modulated by **3** as compared to control. miRNAs are reported as dots located according to fold-variation difference (horizontal axis, > 1.5-fold indicated by the two vertical green lines) and statistical significance of difference calculated by ANOVA (vertical axis, P value < 0.05 log values indicated by the green horizontal line). miRNAs located outside the green lines (i.e. > 1.5-fold and P < 0.05, red dots) are significantly modulated by **3**.

Microarray analysis indicates that treatment with **3** remarkably modified miRNA expression (Fig. 9). Scatter plots highlight that multiple miRNAs changed their expression more than 2-fold in treated cells (vertical axis) as compared to sham-control (horizontal axis), as shown by the many dots located outside the diagonal variation interval indicated by the green lines (Fig. 9). Volcano plot analysis, considering two selection criteria, (i.e. higher than 2-fold variation and statistically significant variation P < 0.05) identified 38 miRNAs whose expression was altered by compound **3**. These miRNAs are involved in a variety of biological functions, as detailed in S.I. Table 1. These findings explain at epigenetic level the activation of apoptotic and necrotic pathways consequent to the high degree of DNA damage induced by **3** in lung cancer A549 cells.

### *In vivo* evaluation of pharmacokinetic and organ distribution

A preliminary analysis of *in vivo* toxicity was conducted using only compound **3** in A/J mice. Since **3** is particularly hydrophobic, a mixture containing 10% DMSO in olive oil was selected as a suitable solvent, after testing its solubility in various means, for administration to the animal. Five mice received solvent alone (sham control) and five mice solvent plus compound **3**. The amount of solvent administered was identical for sham and drug-treated mice. Increasing amounts of **3** (vide infra) could be administered using this solvent without exceeding the previously reported^28,29^ safe limit for DMSO of 1 ml/Kg, in fact no adverse effects were observed for either sham or treated mice. Computational simulations^30^ indicated for compound **3** a rodent oral lethal dose (LD50) of 920 mg/kg. In order to experimentally determine an LD50 in mice, we injected subcutaneously a first dose of 5 mg/Kg which was then increased 5-fold up to 50-fold, with no visible effects on the animals, in line with the computational simulation. Finally, we changed administration from subcutaneous to intraperitoneal, again without detecting any apparent effects. After five days the animals were sacrificed, and the distribution of the compound within the various compartments was determined by HPLC/MS analysis. Whole cell homogenate was used for the analysis after cell debris removal. Under these conditions, cell homogenates include the cellular cytoplasmic fraction plus the nuclear fraction.

Dorsal subcutaneous tissue, spleen, omentum, heart, liver, brain, lung and kidney were removed and homogenised. Concentrations of **3** in tissues/organs were determined by evaluating the area of the extracted ion current at m/z 546.31, corresponding to the compound under analysis [M-H]^−^. The highest value was found in the omentum (1,237,999), a high value was observed at the injection site (532,875) and in the spleen (196,233). Lower concentrations were found in the kidneys (120,117), lungs (32,514) and heart (29,443). Low levels were found in the liver (11,293) and brain (7,735), showing that **3** is able to pass the blood brain barrier (BBB). Thus the amount of **3** found in the brain accounts for only 0.3%, but this value corresponds to ca. 2% if referred to the sum of the amounts found in target organs alone (S.I. Fig. 1).

## Discussion

Structures **3** and **5** were identified as interesting candidates to expand our knowledge on the biological activity of calixpyrroles with a view to further biological studies on more complex bis- and tris-calixpyrrole systems^20,21^. We were expecting to be able to rationalize any biological activity observed for these compounds either in terms of their properties as ionophores or as modulators of growth on cells expressing the GPR30 receptor^22^. After exposure to **3** and **5**, cancer cell lines die by apoptosis after 24 to 48 h, as demonstrated by time course and Annexin V tests. While this type of cell death would be compatible with the disruption of cell homeostasis due to **3** and **5** acting as chloride ionophores, the isolation of DNA covalent adducts and the numerous and considerable changes in miRNA expression for A549 cells exposed to **3** provide evidence for genetic damage to be the likely primary cause of the observed cytotoxicity. This action is rather selective: **3** and **5** showed an EC50 < 5μM when tested on A549 and H727 cell lines but they were less effective against SKOV3 cell lines and were almost ineffective against U87MG Glioma astrocytoma cells.

The small library of compounds selected for testing enables us to conclude that both the calixpyrrole and one *meso*-N-acetamido-substituent are necessary for the observed cytotoxicity. Although we cannot exclude that some other *meso*-substituents could produce cytotoxic molecules, we can conclude that some (e.g. *meso-*4 or *meso-*3*-*aminophenyl, present in **2** and **4** where the acetyl residue is missing) do lead to inactive compounds (at least against the cell lines tested in this study).

A change in the position (*meso-*4 or *meso-*3-aminoacetyl) unit (see **3** *versus* **5**) does not cause dramatic changes in cytotoxicity, but the replacement of the calixpyrrole with a ‘half calix moiety’ produces the inactive compounds **7** and **8**. The lack of activity found for compound **6** indicates that the presence of additional acetanilide units on the calixpyrrole frame does not produce a more active compound, although we cannot exclude that other isomers of **6** might be active. The relative chloride affinities of **3** and **6** in acetonitrile were evaluated by means of ^1^H NMR spectroscopy, and differences were marginal^31^. This result is compatible with chloride transport not being the key to explain the different activities of **3** and **6**. The comparative evaluation of the biological activities of the compounds in Fig. 1 enabled us to conclude that the cytotoxicity of **3** and of **5** are both due to the combined role of three structural elements: i) a single acetanilide unit at ii) a *meso*-position of iii) a complete calix[4]pyrrole structure.

The ability of calixpyrrole derivatives to bind phosphate via multiple hydrogen-bonds (including when this is part of a nucleotide) has been documented previously for a number of analogues of **3** or **5**^32,33^. However, the observed DNA adducts were covalent, which could be ascribed to the reactivity of the acetanilide portion. Indeed, aromatic amines and several of their derivatives are known to form covalent bonds with DNA nucleobases^34,35^. This hypothesis is also consistent with the previously reported ability of acetanilide derivatives to form covalent DNA adducts, as in the study by Rogers on paracetamol^36^, where the ^32^P postlabelling technique was also used. The crucial role of the calixpyrrole unit for the cytotoxicity of **3** and **5** is proven by the lack of activity of compounds **7** and **8**. Therefore, the calixpyrrole component must somehow contribute to the reactivity between DNA and the acetanilide component (presumably as a metabolically activated derivative) of **3** and **5**. We can hypothesise that this could take place via three mechanisms.

One mechanism assumes that the initial non-covalent binding of the calix moiety with phosphate brings the acetanilide unit of the calix into proximity of the nucleobase(s) with a spatial arrangement similar to the one we have proposed previously for the delivery to DNA of cis-Pt(II) complex with calixpyrrole **2**^13^.

A second hypothesis is that some nucleobases might act as ‘guests’ that could be ‘recognised’ by the calixpyrroles **3** and **5**. In a preliminary evaluation of this hypothesis, 1:1 solutions of the DNA nucleobases and **3** in DMSO/acetonitrile were examined by negative ESI-MS. Indeed, adenine and cytosine produced a 1:1 supramolecular complex (see S.I. Figs 3 and 4). If this process were occurring, one would expect the formation of the covalent adducts to take place when the double helix unwinds and the strands separate for replication. In this phase the nucleobases would be ‘free’ to be complexed by the calixpyrrole, this event being followed by an ‘intra-complex’ alkylation of the nucleobase by the acetanilide component.

A third mechanism could be based on the potential for **3** and **5** to act as minor groove binders. To test this hypothesis we performed *in silico* docking experiments in which the calixpyrrole unit of **3** was constrained in the cone conformation typically observed in anionic complexes with phosphates and the *meso*-p-acetanilide unit was pointing either equatorially or perpendicularly with respect to the macroring mean-plane. The modelling indicated the potential of **3** to act as a minor groove binder that places the amide very close to an adenine N3 atom (3.7 Å, see S.I. Fig. 2). Similar results were obtained by constraining the calixpyrrole unit in a 1,3-alternate conformation. This type of interaction is reminiscent of the ability of other polypyrrole-imidazole polyamides^37^ and polypyrrole amides^38^ to act as DNA minor groove binders^39^. These have been shown to enhance the cytotoxic potency of DNA alkylating agents when covalently linked to the pyrrole oligomer^37^, this effect being consistent with the intercalator playing a role in the active delivery of the alkylating moiety to DNA. Although the structural similarity between polypyrrole-imidazole polyamides or polypyrrole amides and calixpyrroles **3** and **5** is limited, the *in silico* experiments suggest that the calixpyrrole unit may be involved in the active delivery of the (presumably metabolically activated) alkylating acetanilide component present in their structure via preliminary minor groove binding. Therefore the potential for **3** to be a minor groove binder, as indicated by the molecular docking simulation, combined with the known mechanism by which aromatic amides form DNA adducts^34–36^, suggests that in **3** (and also in **5**) two portions of the molecule act synergically to produce the genotoxic damage that leads to cell death.

We also hypothesise that the lack of activity of **6** might be due to the enhanced steric hindrance of the two *p*-acetanilide units that could prevent its insertion (or that of an ‘activated’ metabolite) into the DNA minor groove. If metabolic activation is a key step, **6** might lack activity due to it not being a substrate for the activation process.

Finally, one cannot exclude that a combination of these three mechanisms could be operating: the initial step could be phosphate binding, followed by insertion in the minor groove, with nucleobase binding and intra-complex alkylation as the last step. Although the precise mechanism by which **3** and **5** form adducts with DNA has still to be uncovered, a survey of the literature provides good support for the hypotheses described above.

Calixpyrrole **3** not only caused DNA damage (formation of adducts and fragmentation), but also induced epigenetic alterations (miRNA). Taken together these effects provide further information on the pro-apoptotic effect of **3**. MiRNA expressions are highly sensitive to exposure to genotoxic agents^40^ by a process involving the binding of electrophilic metabolites to miRNA precursors in cytoplasm and the alteration induced in the nucleophilic catalytic pocket of DICER^41^. The functions of altered miRNA (Table S1I) shed light on the mechanisms triggered by **3** to kill cancer cells. Indeed, the majority of modulated miRNAs are involved in cell cycle blockage and apoptosis activation. Two modulated miRNAs (miR-34, miR-660) are directly linked to the P53 pathway, this indicating that apoptosis is triggered as a consequence of DNA damage. General anti-oncogenic activity is envisaged for many modulated miRNAs including oncogene suppression, TGF inhibition, blockage of EMT transition, cell migration invasion and metastatisation. Overall, these data provide evidence of the specific anti-cancer activity deployed at epigenetic level by calixpyrrole **3**.

The pharmacokinetic study was limited to **3** because this was found to be the most active of the tested compounds against the highly aggressive A549 lung cancer cells. Our interest in this was motivated by the fact that lung cancer represents the most frequently occurring form of this disease and it is also the most common cause of death from cancer worldwide. The prognosis for lung cancer is poor^42^; in most countries there is a 5-year survival rate of 10%. One of the reasons for such a low survival rate is the ability of lung cancer to give rise to metastases affecting mainly the brain, as well as other organs^43^. Primary and secondary brain tumours are particularly difficult to manage due to the BBB hampering drug penetration. The calix[4]pyrrole derivatives selected in this study are highly lipophilic, this being an important prerequisite to pass the BBB^44^.

The method used for the bio-distribution studies gave the ratios of compound **3** in the different tissues. These proportions were determined by using equal weights of the different tissues that were analysed by the same method, therefore the ion currents measured for the MS peak of **3** in the different tissues could be compared directly. Compound **3** is highly lipophilic, and it is not surprising that it accumulates in the omentum, while the high concentration at the injection point is also explained by its low solubility in aqueous medium. The proportion of **3** reaching the target organs was only 18% of the total quantity found in the various tissues taken together. This 18% was distributed in the organs as shown in Fig. S1. The omentum may act as a natural reservoir for the administered calixpyrrole **3**. In this preliminary study we did not investigate how the biodistribution changes over time or upon the loss of fatty tissue by an animal affected by a developing cancer. As regards the ‘mobile’ fraction of **3**, significant amounts reach the lungs (8%) and the brain (2%). This is particularly interesting because of the highly effective activity of **3** against the A549 lung cancer cells. The ability to cross the BBB is of great importance since brain metastases are common evolutions of lung cancers that are extremely difficult to treat^43^. Therefore, the observed biodistribution data for **3** are of great interest. The *in vivo* study also indicated no apparent short-term toxic effects even at very high dosages (up to 250 mg/Kg).

In conclusion, the results described herein indicate that **3** and **5** can be considered lead structures for the development of novel potentially selective anticancer drugs that can be tolerated *in vivo*^45^.

## Materials and Methods

### General procedure

Solvents were dried using molecular sieves (4 Å) or by following standard procedures^46^. Pyrrole was distilled before use. All other chemicals were standard reagent grade and were used without further purification. All air and/or moisture-sensitive reactions were conducted under an inert atmosphere. Thin-layer chromatography was carried out on Merck SiO_2_ 60F254 plastic plates. Compounds were visualised with vanillin or by examination under UV light. Column chromatography was conducted using silica gel (Aldrich, 230–400 mesh, 60 Å). ^1^H and ^13^C NMR spectra were recorded on a Varian 500 spectrometer at 500 and 125 MHz, respectively, with the residual proton resonances of the solvent (CD_2_Cl_2_) used as references. NMR solvent was used as supplied in sealed ampoules and care was taken to minimize exposure to moisture. MS spectra were recorded on Agilent 6210 TOF mass spectrometer (Agilent Technologies, Palo Alto, CA, USA) equipped with an electrospray ion source operating in negative polarity for compounds **2**, **5** and **6**, and on a triple quadrupole WATERS TQMS spectrometer operating in positive polarity for **7** and **8**.

Before being used for the biological tests, compounds **2**–**8** were vacuum-dried to remove traces of residual ethyl acetate (EtOAc) from the chromatographic purifications.

#### *meso*-(4-aminophenyl)-*meso*-hepta(methyl)calix[4]pyrrole 2

This compound was prepared as described by us previously^13^.

#### *meso*-(4-acetamidophenyl)-*meso*-hepta(methyl)calix[4]pyrrole 3

A solution of acetyl chloride (31.1 mg, 0.4 mmol) in dry DCM (2 mL) was gradually added to a stirred suspension of **2** (200 mg, 0.4 mmol) and excess K_2_CO_3_ (55.3 mg, 0.4 mmol) in DCM (20 mL), at room temperature, in Ar atmosphere. The reaction was monitored by TLC (SiO_2_, DCM) until complete disappearance of the starting material (30 min.). The reaction mixture was extracted with water (1 × 15 mL) and with a saturated solution of NaHCO_3_ (1 × 15 mL). The organic phase was dried (MgSO_4_) and concentrated under reduced pressure. The crude was subjected to column chromatography (SiO_2_, DCM, DCM/EtOAc, 9:1) to give **2** (108 mg. 49%, m.p. < 250 °C from EtOAc, dec.) as a white solid which appeared pure by TLC, ^1^H and ^13^C NMR analyses and ESI-MS analyses. ^1^H NMR (500 MHz, CD_2_Cl_2_) δ 1.48 (s, 3 H, CH_3_), 1.50 (s, 6 H, CH_3_), 1.52 (s, 3 H, CH_3_), 1.56 (s, 6 H, CH_3_), 1.85 (s, 3 H, CH_3_), 2.11 (s, 3 H, COCH_3_), 5.65 (m, 2 H, pyrrole β-CH), 5.87 (m, 4 H, pyrrole β-CH), 5.91 (m, 2 H, pyrrole β-CH), 6.91 (AB system, 2 H, Ar-CH), 7.13 (sb, 2 H, pyrrole-NH), 7.23 (sb, 1 H, NHCO), 7.27 (sb, 2 H, pyrrole-NH), 7.34 (AB system, 2 H, Ar-CH); ^13^C NMR (125 MHz, CD_2_Cl_2_) δ 24.6, 27.6, 27.8, 28.2, 29.9, 30.1 (CH_3_), 35.3, 35.3, 44.5 (Cq), 103.0, 103.0, 103.3, 106.1 (pyrrole-CH), 119.2, 128.1 (Ar-CH), 136.7, 137.7, 138.6, 138.8, 139.2, 144.0 (Cq), 168.4 (CO). Calculated for C_35_H_41_N_5_O 547.33111; found (ESI-MS) [M-H]^−^ 546.3106, the calculated and observed isotopic patterns were in good agreement.

#### *meso*-(3-aminophenyl)-*meso*-hepta(methyl)calix[4]pyrrole 4

This compound was prepared as described by us previously^20^.

#### *meso*-(3-acetamidophenyl)-*meso*-hepta(methyl)calix[4]pyrrole 5

This compound was prepared by N-acetylation of compound **4** as described for **3**. (including the same quantities): (46%, 100 mg, m.p. < 250 °C from EtOAc, dec.); ^1^H NMR (500 MHz, CD_2_Cl_2_) δ 1.49 (s, 9 H, CH_3_), 1.52 (s, 3 H, CH_3_), 1.56 (s, 6 H, CH_3_), 1.87 (s, 3 H, CH_3_), 2.07 (s, 3 H, COCH_3_), 5.72 (m, 2 H, pyrrole β-CH), 5.88 (m, 4 H, pyrrole β-CH), 5.92 (m, 2 H, pyrrole β-CH), 6.72 (d, 1 H, Ar-CH), 7.04 (s, 1 H, Ar-CH), 7.19 (t, 1 H, Ar-CH), 7.23 (sb, 1 H, NHCO), 7.34 (sb, 2 H, pyrrole-NH), 7.35 (d, 1 H, Ar-CH), 7.48 (sb, 2 H, pyrrole-NH); ^13^C NMR (125 MHz, CD_2_Cl_2_) δ 24.5, 27.9, 27.9, 28.3, 29.9, 29.9 (CH_3_), 35.3, 35.4, 44.9 (Cq), 103.0, 103.0, 103.1, 105.8 (pyrrole-CH), 118.8, 120.1, 123.9, 128.5 (Ar-CH), 136.5, 137.6, 138.7, 138.9, 139.3, 149.3 (Cq), 168.6 (CO). Calculated for C_35_H_41_N_5_O 547.33111; found (ESI-MS) [M-H]^−^ 546.3111, the calculated and experimental isotopic patterns were in good agreement.

#### (10α,20α)-10,20-bis(4-acemidophenyl) 5,5,10,15,15,20-hexamethy-calix[4]pyrrole 6

This compound was obtained by bis N-acetylation of the previously reported^23^ (10α,20α)-10,20-bis(4-acemidophenyl)5,5,10,15,15,20-hexamethy-calix[4]pyrrole as described for **3**, doubling the amounts of acetylchloride and K_2_CO_3_. **6**: (47% 125 mg, m.p. < 250 °C from EtOAc, dec); ^1^H NMR (500 MHz, CD_2_Cl_2_) δ 1.51 (s, 6 H, CH_3_), 1.62 (s, 6 H, CH_3_), 1.86 (s, 6 H, CH_3_), 2.10 (s, 6 H, COCH_3_), 5.63 (m, 4 H, pyrrole β-CH), 5.92 (m, 4 H, pyrrole β-CH), 6.89 (AB system, 4 H, Ar-CH), 7.21 (sb, 2 H, NHCO), 7.30 (sb, 4 H, pyrrole-NH), 7.34 (AB system, 4 H, Ar-CH); 13 C NMR (125 MHz, CD_2_Cl_2_) δ 24.4 (COCH_3_), 26.9, 27.4, 30.0 (CH_3_), 34.9, 44.2 (Cq), 103.1, 105.8 (pyrrole-CH), 118.8, 127.8 (Ar-CH), 136.4, 138.4, 143.8 (Cq), 168.1 (CO). Calculated for C_42_H_46_N_6_O_2_ 666.36822; found (ESI-MS) [M-H]^−^ 665.3571.

#### 5-Methyl-5-(4-acetamidophenyl)dipyrromethane 7

This compound was prepared by N-acylation of the previously reported^13^ 5-Methyl-5-(4-aminophenyl)dipyrromethane, as described for **3**.

**7**: (80%); ^1^H NMR (500 MHz, CD_2_Cl_2_) δ 2.01 (s, 3 H, CH_3_), 2.08 (s, 3 H, COCH_3_), 5.94 (m, 2 H, pyrrole β-CH), 6.11 (m, 2 H, pyrrole β-CH), 6.65 (m, 2 H, pyrrole α-CH), 7.02 (AB system, 2 H, Ar-CH), 7.34 (AB system, 2 H, Ar-CH), 7.44 (sb, 1 H, NHCO), 8.06 (sb, 2 H, pyrrole-NH); ^13^C NMR (125 MHz, CD_2_Cl_2_) δ 24.4, 28.9 (CH_3_), 44.5 (Cq), 106.3, 108.1, 117.4 (pyrrole-CH), 120.2, 128.8 (Ar-CH), 136.7, 137.7, 143.8 (Cq), 168.7 (CO). Calculated for C_18_H_19_N_3_O 293,15281; found (ESI-MS) [M + H]^+^ 294.22, the calculated and experimental isotopic patterns were in good agreement.

#### 5-Methyl-5-(3-acetamidophenyl)dipyrromethane 8

This compound was prepared by N-acylation of 5-Methyl-5-(3-aminophenyl)dipyrromethane^20^ as described for **3**.

**8**: **(**80%); ^1^H NMR (500 MHz, CD_3_CN) δ 1.95 (s, 3 H, CH_3_), 1.98 (s, 3 H, COCH_3_), 5.80 (m, 2 H, pyrrole β-CH), 6.00 (m, 2 H, pyrrole β-CH), 6.64 (m, 2 H, pyrrole α-CH), 6.74 (d, 1 H, Ar-CH), 7.15 (s, 1 H, Ar-CH), 7.19 (t, 1 H, Ar-CH), 7.50 (d, 1 H, Ar-CH), 8.21 (sb, 1 H, NHCO), 8.69 (sb, 2 H, pyrrole-NH); ^13^C NMR (125 MHz, CD_3_CN) δ 23.9, 28.6 (CH_3_), 45.4 (Cq), 106.6, 107.9, 117.7 (pyrrole-CH), 118.0, 119.4, 123.3, 128.9 (Ar-CH), 138.2, 139.6, 149.6 (Cq), 169.2 (CO). Calculated for C_18_H_19_N_3_O 293,15281; found (ESI-MS) [M + H]^+^ 294.12, the calculated and experimental isotopic patterns were in good agreement.

### Cell culture

Human lung non-small cell carcinoma H727 and SKOV-3 ovary cancer cell lines were purchased from ATCC (Manassas, VA) and cultured respectively in RPMI (Pan-Biotech, Aidenbach, Germany) and DMEM (Sigma-Aldrich, Milan, Italy), both supplemented with 10% foetal calf serum (Euroclone, Milan, Italy), 2 mM L-glutamine (Euroclone, Milan, Italy) and 1% Penicillin-Streptomycin (Euroclone, Milan, Italy) at 37 °C in a 5% CO_2_ incubator. A549 human lung adenocarcinoma, U87MG glioblastoma, MCF7 (ER + ) and MDA-MB-231 (ER-triple negative) human breast cancer cell lines were purchased from the BBCF (Biological Bank and Cell Factory, IRCCS Policlinico San Martino, Genoa, Italy). They were cultured in DMEM (Sigma-Aldrich, Milan, Italy), supplemented with 10% foetal calf serum (Euroclone, Milan, Italy), 2 mM L-glutamine (Euroclone, Milan, Italy) and 1% Penicillin-Streptomycin (Euroclone, Milan, Italy) at 37 °C in a 5% CO_2_ incubator.

### Time-course experiment

A time-course experiment was performed by treating the A549 cell line with the lowest dose (5 μM) of compounds **3** and **5**. The treatment was blocked after 3, 6, 12 and 24 hours. The cells were then fixed with Formalin, coloured with crystal violet and the results were read on a microplate photometer (Multiskan FC, Thermo Scientific) at 570 nm.

### Pharmacokinetic studies with liver metabolic fraction (S12)

The following working solutions were prepared:

- 10 μM containing **3** or **5** *with* S12: a solution of **3** or **5** in DMSO (5 mM, 1μL) was diluted 1:500 with the following mixture: deionised water (33.2% *v/v*), phosphate-buffered saline (50% *v/v*, PBS, Euroclone, Milan, Italy), nicotinamide adenine dinucleotide phosphate (4% *v/v*, NADP, Sigma-Aldrich, Milan, Italy), glucose 6-phosphate (0.5% *v/v*, G6P, Sigma-Aldrich, Milan, Italy), MgCl_2_ (2% *v/v*, BDH, VWR International, Milan, Italy) and S12 (10.3% *v/v*);
- 10 mM containing **3** or **5** *without* S12: a solution of **3** or **5** in DMSO (5 mM, 1μL) was diluted 1:500 with the following mixture: deionised water (43.5% *v/v*), phosphate-buffered saline (50% *v/v*, PBS, Euroclone, Milan, Italy), nicotinamide adenine dinucleotide phosphate (4% *v/v*, NADP, Sigma-Aldrich, Milan, Italy), glucose 6-phosphate (0.5% *v/v*, G6P, Sigma-Aldrich, Milan, Italy), MgCl_2_ (2% *v/v*, BDH, VWR International, Milan, Italy),
- Sham test solution *with* S12: DMSO (1 μL) was diluted 1:500 as indicated for the solutions of **3** or **5** *with* S12;
- Sham test solution *without* S12: DMSO (1 μL) was diluted 1:500 as indicated for the solutions of **3** or **5** *without* S12.

A549 cell line was seeded in 96-microwell flat-bottom plates at a density of 6 × 10^3^ cells per well in 100 μL of culture medium DMEM (Sigma-Aldrich, Milan, Italy). The next day, cells were treated with 100 μL of the working solutions, obtaining a 5 μM concentration for tests with **3** or **5** and identical content of DMSO (0.1%) in all cases, including sham tests. The solutions were incubated at 37 °C for 30 min. Both native functional S12 fraction and heat-inactivated (60 °C for 1 h) S12 were tested. Treatment time was 12 h, followed by the MTT test as described below.

### Cell viability assays

To evaluate cell viability in response to chemical compound treatments, we performed MTT assays on lung, glioma-astrocytoma, breast and ovarian cancer cell lines. For this purpose a 5 mM stock solution of each compound was prepared in neat DMSO (Sigma-Aldrich, Milan, Italy) and this was then diluted with foetal bovine serum (Biosigma, Cona, Venice, Italy) in the following proportions: 1:50, 3:250, 1:250 and 1:500. Working solutions having the following concentrations of compound and DMSO were thereby obtained: (100 μM, 2% DMSO), (60 μM, 1,2% DMSO), (20 μM, 0.4% DMSO), (10 μM, 0.2%). Adding 100 μL of the above working solutions to 100 μL of cell culture (as described below) resulted in a further 1:2 dilution and produced the concentrations 50 μM, 30 μM, 10 μM, and 5 μM (indicated in the viability plots of Fig. 3), containing 1%, 0.6%, 0.2% and 0.1% DMSO respectively. Sham tests were conducted using identical concentrations of DMSO solvent alone.

Cells were seeded in 100 μl of medium at a concentration per well to reach ca. 80% of confluence in the untreated wells at the end of the assays. The next day, 100 μL of culture medium containing different concentrations (10 μΜ–100 μΜ) of the tested compounds was added to cells. The cells were further incubated for 24 h. On the third day, 20 μl of MTT stock solution (2 mg/ml in PBS) was added for an additional 4 h of incubation. At the end of this incubation time, the precipitated formazan was dissolved in 100 μL of DMSO. After 20 min in the dark, the results were read on a microplate photometer (Multiskan FC, Thermo Scientific, Waltham, Massachusetts) at 570 nm and normalised with respect to the corresponding sham test (results are expressed as percentage of the control samples). Each test was repeated three times in eight replicates and means and standard deviations calculated.

### Annexin-V apoptosis assay

A549 cells, seeded at a density of 2 × 10^5^ cells per well in 1 mL of culture medium DMEM (Sigma-Aldrich, Milan, Italy), were treated with compounds **3** and **5** at a concentration of 5 μM for 24 h. Muse™ Annexin V & Dead Cell Assay was then performed. Cells were dissociated from each well to obtain single-cell suspensions and 100 μL of these suspensions was added to each tube together with 100 μL of the Muse™ Annexin V & Dead Cell Reagent (BD Biosciences Pharmingen 2350 Qume Drive San Jose, California, USA). The samples were mixed thoroughly by vortexing at a medium speed for 3 to 5 seconds and were then stained at room temperature in the dark for 20 min, before being analysed by flow cytometry (FACS Canto II cytometer, Becton Dickinson BD).

### Comet assay

A549 cells were seeded in 6-well flat-bottom plates at a density of 2 × 10^4^ cells per well in 1 mL of culture medium DMEM (Sigma-Aldrich, Milan, Italy) and treated with compounds **2**–**5** (5 μM). After 24 h, the cells were dissociated using trypsin (Euroclone), centrifuged at 1000 rpm x 5 min, suspended in 1% (w/v) low-melting-point agarose at a concentration of 1 × 10^4^ cells/mL and applied to the surface of a microscope slide to form a microgel and allowed to set at 4 °C for 5 min. The slides were submerged overnight at 4 °C in cell lysis buffer solution (2.5 M NaCl, 100 mM EDTA, 10 mM Tris-HCl, pH 10) to which 1% Triton X-100 and 10% DMSO were added before use. An alkaline solution (0.3 M NaOH, 1 mM EDTA, pH 13) was prepared to perform DNA unwinding and electrophoresis at 300 mA and 25 V. After the neutralisation step, the slides were dehydrated with absolute ethanol. Analyses of samples were carried out on slides stained with ethidium bromide (2 μg/ml in H_2_O) using a fluorescence microscope at 200X magnification (Leica Microsystems, Mannheim, Germany) equipped with a digital camera. Images of at least 100 randomly selected nuclei were acquired and analysed using an automated imaging system CASP or Comet Assay (SoftwareProject, http://www.casp.sourceforge.net). DNA damage (alkali–labile sites and both double- and single-stranded DNA breaks) was quantified in terms of % tail DNA. All results are expressed as mean ± SD. The statistical significance was evaluated by ANOVA and Student’s t test^47^.

### DNA adducts

A549 cells, grown in two 75 cm^2^ flasks, were treated for 24 h, one with compound **3** and the other with **5**, both at the concentration of 5 μM. A third flask with A549 cells was used as control. The cells were then detached and DNA was extracted using the GenElute Mammalian Genomic DNA Miniprep Kit following the manufacturer’s instructions (Sigma-Aldrich, Milan, Italy). Aliquots of 6 μg DNA were assayed, after butanol extraction, to evaluate the presence of adducts by ^32^P postlabelling^27,48^.

### MicroRNA expression

A549 total RNA was purified from the supernatant using a commercially available kit (miRNeasy, Qiagen, Valencia, CA, USA) by adding 100% ethanol and centrifuging at 8000 × *g*. The spin columns were washed twice, and the trapped RNA was eluted using ultrapure water (80 μl). The amounts and purity of extracted RNA were evaluated by fiber optic spectrophotometer (Nanodrop ND-1000, Thermo Scientific, Wilmington, DE, USA). To evaluate the expression of miRNAs, the 7th generation miRCURY LNA™ microRNA Array (Exiqon, Vedbaek, Denmark) was used, which contains 3100 capture probes covering human, mouse, and rat. This microarray analyses the expression of 1928 human miRNAs. The total RNA from each sample was labelled using Label IT® miRNA Labelling Kits, version 2 (Mirus Bio, Madison, WI, USA) following the standard protocol. Total RNA (500 ng) was mixed with 10 μl labelling buffer, 4 μl Label IT reagent (containing Cy3 or Cy5 fluorescent tracers), and water 86 μl. The samples were incubated at 37 °C for 1 h and the reaction stopped by adding 10 μl Stop Reagent. Differently labelled samples (with Cy3 and Cy5, respectively) were purified by column chromatography in 25 μl elution buffer. Hybridisation solution (2 × 25 μl, EXIQON) was then added and mixed thoroughly, and the resulting mixture denatured at 65 °C for 3 min. The labelled mix was transferred to the microarray and covered with coverslips. The hybridisation was performed in GlassArray Hybridization Cassettes (Invitrogen Ltd, Paisley, UK) in a water bath at 37 °C for 16 h and then a wash sequence performed. The array was dried by centrifugation and scanned by a laser scanner (ScanArray, PerkinElmer, Waltham, MA, USA) to record fluorescent signals produced by each spotted probe effectively hybridised with the corresponding miRNA^49^.

### *In vivo* study

Based on the observed efficacy *in vitro*, compound **3** was chosen for preliminary *in vivo* studies. The main challenge was to find a non-toxic solvent for mice in which this compound could be dissolved. We therefore performed solubility tests using various solvents, including olive oil, propylene glycol, aqueous solution of sodium chloride 2 M and dimethyl sulfoxide (DMSO). Suspensions of compound **3** were obtained using each of these solvents alone, except for DMSO, and also heating the solutions. Finally, we achieved solubility using 10% DMSO in olive oil. A/J mice were used for the *in vivo* studies. The Web Server PROTOX^30^ predicted an oral LD50 of 920 mg/kg in rodents for **3** and this was used to determine the amount of compound to be administered. Since we decided to inject the solution of **3** subcutaneously, we started with a low dose (5 mg/Kg), subsequently increasing the amount 5- and 50-fold and we also changed administration from subcutaneous to intraperitoneal.

All treatments were performed in line with the ethical guidelines for animal experiments of the European Community Directive 2010/63/UO 22/09/2010 and the Guide for the care and use of laboratory animals 8^th^ edition, The US National Academic Press 2010. The experimental protocol was approved by the Institute of Cell Biology and Biotechnology, L.N. Gumilyov Eurasian National University, Astana, Kazakhstan.

### Biodistribution of 3

To measure the distribution of compound **3** in different organs of mice, we performed a preliminary analysis using HPLC-MS. Mice were subcutaneously injected with 250 mg/Kg of compound **3** and five days later organs (lung, brain, spleen, omentum, kidney, liver, heart) were harvested as well as dorsal subcutaneous tissue from the injection site. Identical amounts (87 mg) were used for HPLC/MS. The protocol^50^ requires that each sample is suspended in water (10 μl/mg) and homogenised for 3 min at 30 s^−1^ using TissueLyser (Qiagen, Hilden, Germany). Ethanol was then added to each sample (1:2 homogenate:ethanol), vortexed, centrifuged (13,200 rpm, 10 min) and the supernatant was collected. This step was repeated using equal volumes of ethanol and homogenate. The supernatants were stored at −80 °C. The amount of **3** was determined by HPLC-ESI/TOF-MS, operating in negative ion mode, using an Agilent 1200 series chromatographic system, equipped with G1379B degasser, G1376A capillary pump, and G1377A autosampler. Each cryopreserved (1900 μL) sample was centrifuged (14,000 rpm, 15 min) and vacuum-dried in SpeedVac overnight. Pellets were resuspended in acetonitrile (100 μL), sonicated for 5 min and centrifuged (14,000 rpm, 10 min). Each sample (3 μL) was injected onto a 1.0 mm × 150 mm, 300 Å pore size, 3.5 μm particle size Symmetry 300 C18 column (Waters Corp., Milford, MA). The eluents were 95% water – 5% acetonitrile (eluent A) and 5% water – 95% acetonitrile (eluent B), both containing 0.1% formic acid. The flow rate was 20 μl/minute and the elution was performed at 25 °C. The mobile phase was: isocratic 50% B for 10 min followed by a linear gradient from 50% to 100% B in 20 min, maintained at 100% B for 10 min and, finally, a linear gradient to 50% B in 2 min. The re-equilibration time in 50% B was 10 min. After HPLC separation, the eluent was sent to an Agilent 6210 TOF-MS equipped with an electrospray ion source. Negative full-scan mass spectra were recorded using Agilent Mass Hunter software in the mass range of m/z 100–1000. The following operational parameters were applied: capillary voltage: 3000 V; nebulizer pressure: 20 psig; drying gas: 5 L/min; gas temperature: 300 °C; fragmentor voltage: 3500 V; skimmer voltage: 60 V; octapole RF: 250 V. The full scan data were processed using Agilent Mass Hunter Qualitative Analysis, ver. B.02.00 software. The amount of compound **3** was measured from the extracted ion current (EIC) peak area (EIC m/z 543.31 [M−H]^−^).

### Molecular Docking simulations

Docking simulations were performed using Autodock v.4.2.2.^51^, using the crystallographic structure of a fragment of B-DNA complexed with cis-platin^52^ as target. The program standard default and a search grid encompassing the whole DNA fragment surface were used. The docking experiment consisted of 100 Lamarckian Genetic Algorithm runs. The generated docking poses were ranked in order of increasing docking energy values and clustered on the basis of a RMSD cut-off value of 2.0 Å. The structural analysis of the lowest energy solutions of each cluster, enabled us to identify the binding mode of **3**. The structure was drawn using the program MarvinSketch [ChemAxon Ltd, Budapest, Hu]; The *meso*-acetamidophenyl chain of **3** was handled as a flexible unit while the calix[4]pyrrole moiety was ‘frozen’ in the cone conformation that is normally adopted in calix[4]pyrrole-anion complexes. The double helix was treated as a rigid object. S.I. Fig. 2 was drawn using the program Chimera^53^. Additional docking simulations were conducted locking the calixpyrrole component of **3** in a 1,3-alternate conformation, as found when the calixpyrrole binds neutral guests. Despite the small differences in the pose of the macrocycles within the DNA minor groove, the acetyl moiety of **3** is positioned at a reasonable distance from the N3 atom of an adenine, allowing the possibility of forming a covalent bond to the double helix (S.I. Fig. 2C).

### Data availability

Experimental data are available upon request to the corresponding authors.

## Electronic supplementary material

Supplementary Information
