# Supplementary material for: A novel calix[4]pyrrole derivative as a potential anticancer agent that forms genotoxic adducts with DNA
Source: Sci Rep. 2018 Jul 23;8:11075. doi: 10.1038/s41598-018-29314-9 (PMC6056420; doi:10.1038/s41598-018-29314-9)
Supplement: Supplementary file 1 — Supplementary Information [file 41598_2018_29314_MOESM1_ESM.docx]

**Supplementary Information**

**A novel calix[4]pyrrole derivative as a potential anticancer agent that forms genotoxic adducts with DNA.**

Marta Geretto^1,§^, Marco Ponassi^2,§^, Martina Casale^3^, Alessandra Pulliero^1^, Grazia Cafeo^3^, Ferdinando Malagreca^3^, Aldo Profumo^2^, Enrica Balza^2^, Rakhmetkazhi Bersimbaev^4^, Franz Heinrich Kohnke^3,^*, Camillo Rosano^2,^*, Alberto Izzotti^1,2^

^1^Department of Health Sciences, University of Genova, Italy; ^2^IRCCS Ospedale Policlinico San Martino, Genova, Italy; ^3^ CHIBIOFARAM, University of Messina, Italy ^4^Department of General Biology and Genomics, Institute of Cell Biology and Biotechnology, L.N. Gumyliov Eurasian National University, Astana, Kazakhstan

^§^ M. G. and M. P. contributed equally to this work. ^*^Corresponding authors. Correspondence and requests for materials should be addressed to F.H.K (email: franz@unime.it) or to C.R. (camillo.rosano@hsanmartino.it)

**S.I. Figure 1:** Normalised distribution of **3** in the target organs.


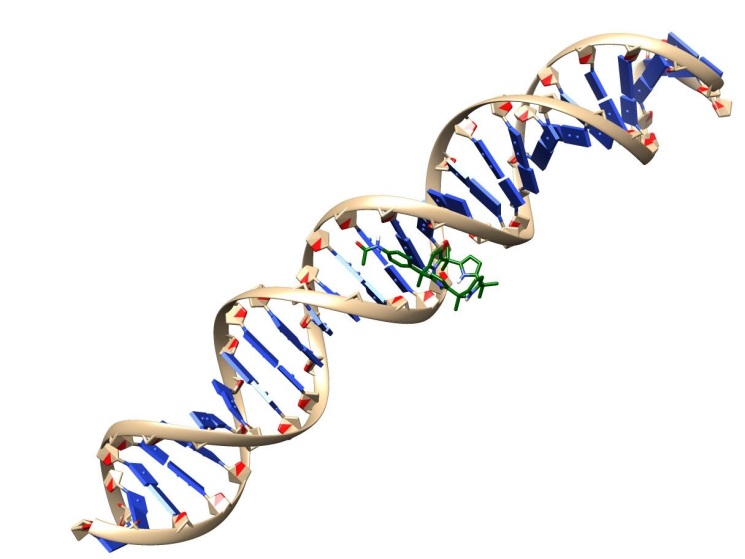

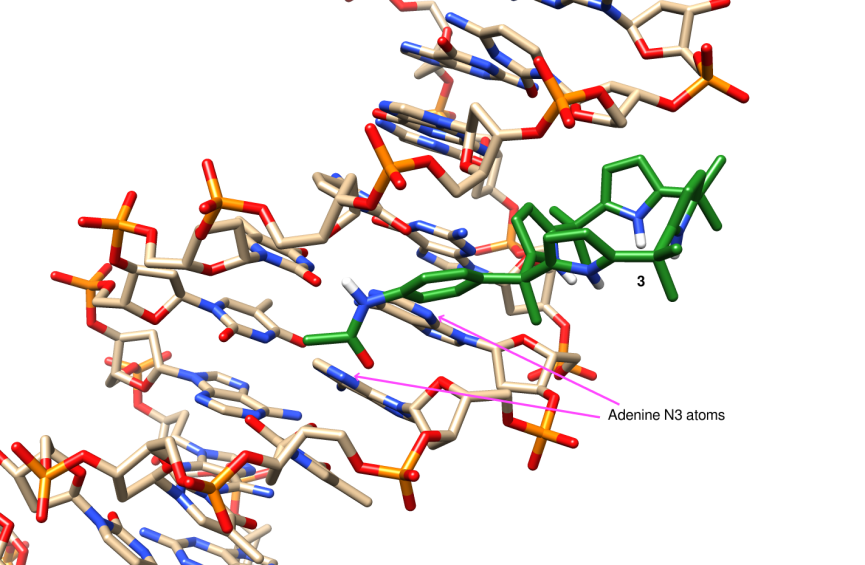


**B**)

**A)**

**
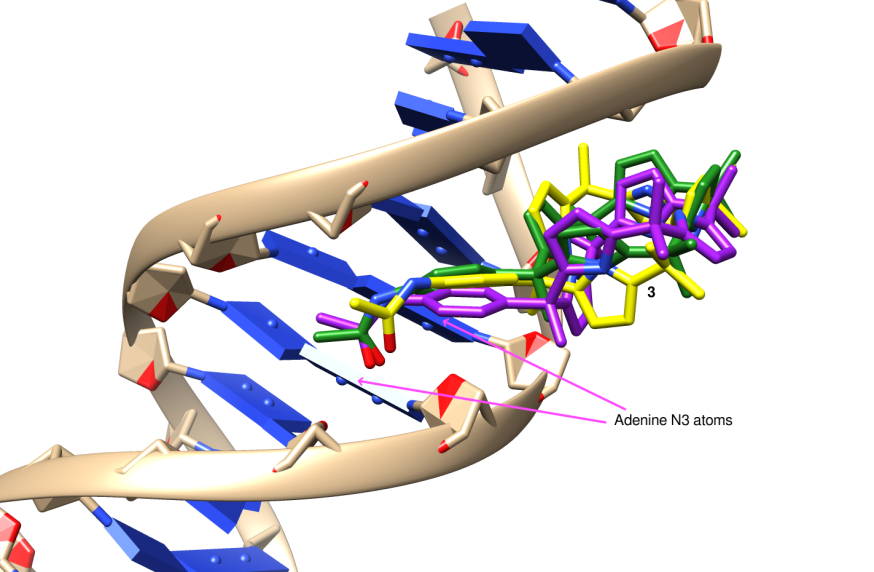
**

**C)**

**S.I. Figure 2** Binding mode of **3** to DNA resulting from molecular docking simulations. Panel **A**) Localisation of **3** (drawn in green sticks) within a DNA fragment (ribbon representation). The square region contoured in dark red is reported in Panel **B**) where two adenine nucleobases close to the acetyl moiety of **3** are visible.

**Formation of Complexes of single nucleobases with 3 investigated by ESI-MS.**

High-resolution analysis was performed on an Agilent 6210 TOF mass spectrometer (Agilent Technologies, Palo Alto, CA, USA) equipped with an electrospray ion source operating in negative polarity. Calixpyrrole **3** was dissolved in DMSO to give a 20 mM solution. Solutions of adenine, cytosine, guanine, thymine and uracil having the same concentration were also prepared in water. Equal volumes of **3** and nucleobase solutions were mixed and diluted with acetonitrile to obtain a concentration 50 μM. Samples were analysed by direct infusion, introducing the sample by means of an automated syringe into the electrospray ion source of the mass spectrometer. The following parameters were applied: capillary voltage: 4500 V; nebuliser pressure: 30 psig; drying gas: 5 L/min; gas temperature: 300 °C; fragmentor: 50 V; skimmer: 60 V; octapole RF: 250 V. Accurate mass spectra were recorded in the range m/z=100–3500. Internal mass calibration was performed automatically by the instrument using a dual-nebuliser electrospray source with an automated calibrant delivery system. The Agilent ESI Tuning Mix (Agilent Technologies) was used as the calibrant solution, and contains the internal reference masses for the positive and the negative ion mode. The full-scan data recorded were processed using Mass Hunter Qualitative Analysis ver. B.02.00 (by Agilent Technologies, Palo Alto, CA, USA). The ion corresponding to the 1:1 supramolecular complex could be detected only for adenine (S.I. Figure 3) and cytosine
(S.I. Figure 4).





**S.I. Figure 3**. ESI/TOF MS of a mixture of Calixpyrrole **3** and Adenine showing the formation of the **3**•Adenine complex (m/z 681.35). The analysis also showed the formation of the **3•**Adenine**•**Chloride complex (m/z 717.38).

**

**

**S.I. Figure 4.** ESI/TOF MS of a mixture of Calixpyrrole **3** and Cytosine showing the formation of the **3**•Cytosine complex (m/z 636.34).


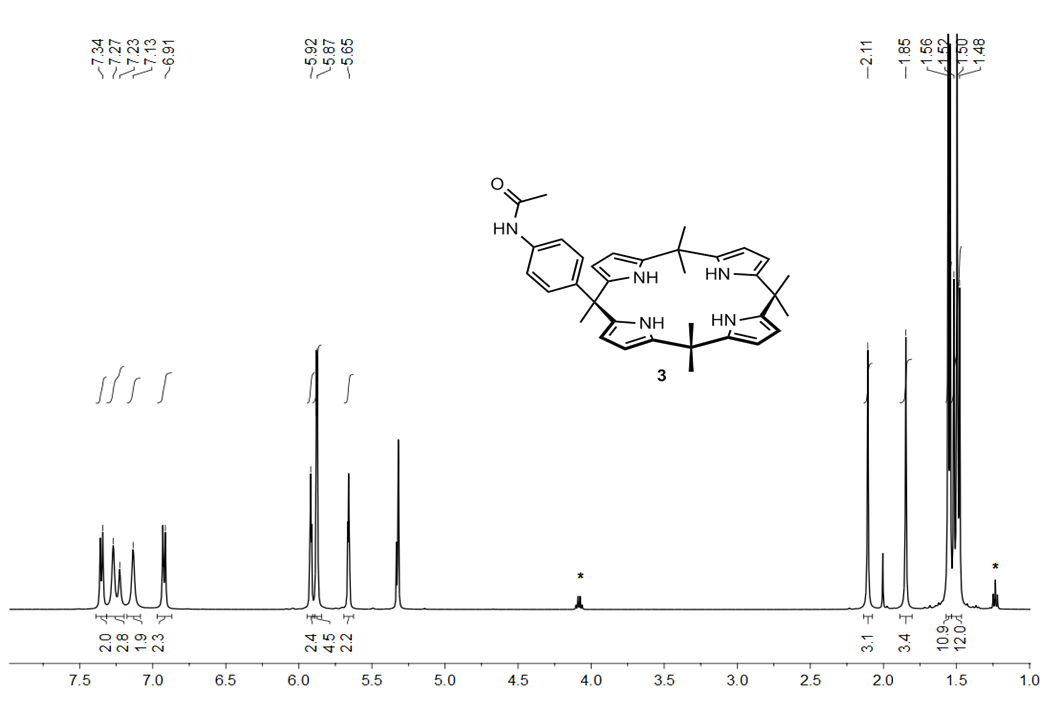


**S.I. Figure 5**. Partial ^1^H NMR (500 MHz, CD_2_Cl_2_,) spectrum of **3**. * Traces of residual EtOAc.


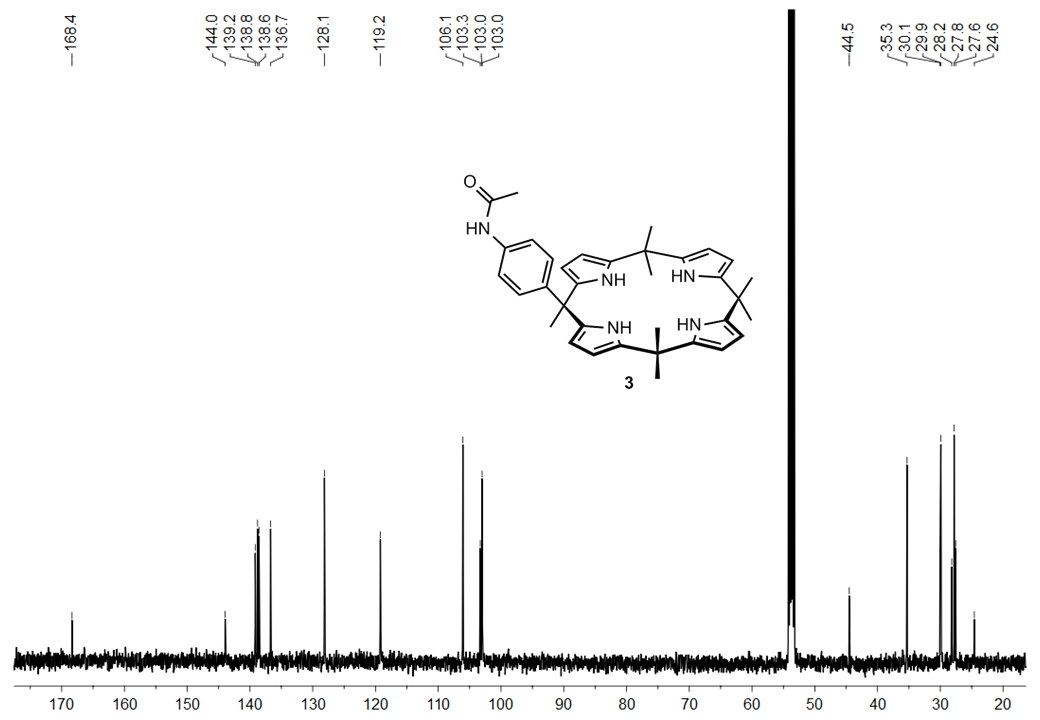


**S.I. Figure 6**. ^13^C NMR (125 MHz, CD_2_Cl_2_,) spectrum of **3**.


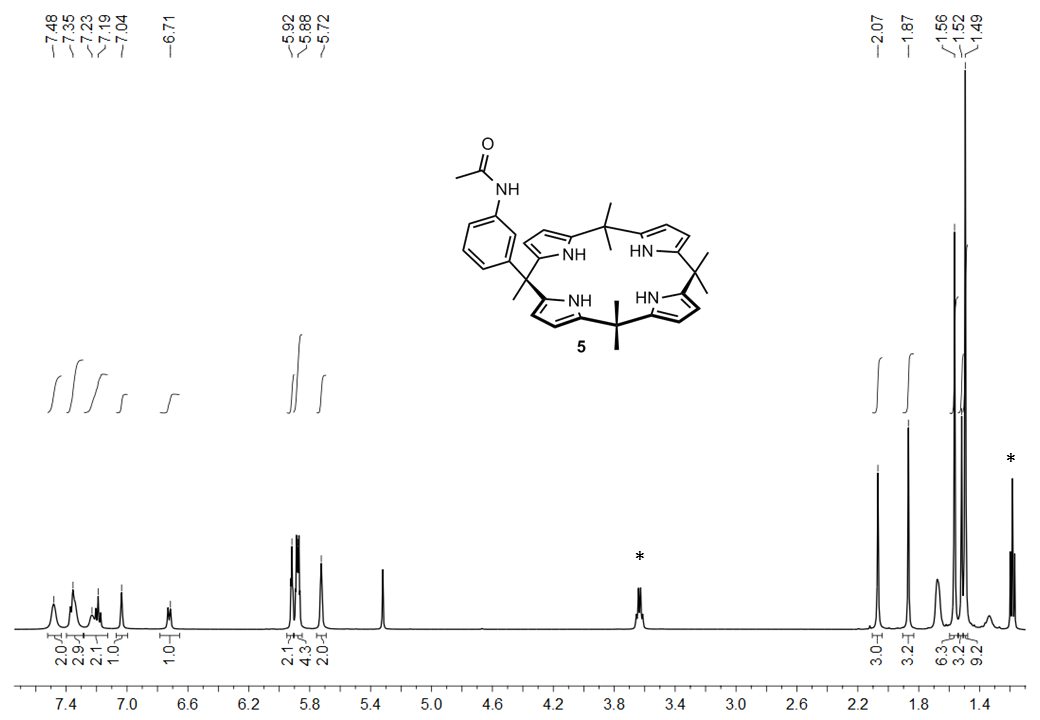


**S.I. Figure 7**. Partial ^1^H NMR (500 MHz, CD_2_Cl_2_,) spectrum of **5**. * Traces of residual EtOAc.


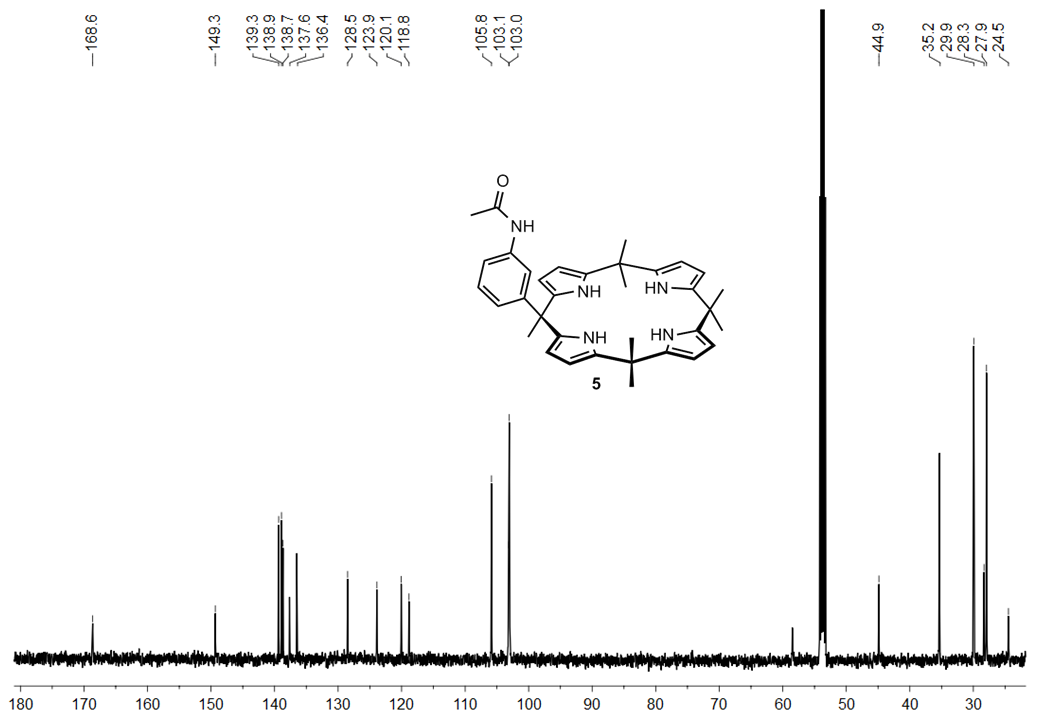


**S.I. Figure 8**. ^13^C NMR (125 MHz, CD_2_Cl_2_,) spectrum of **5**.


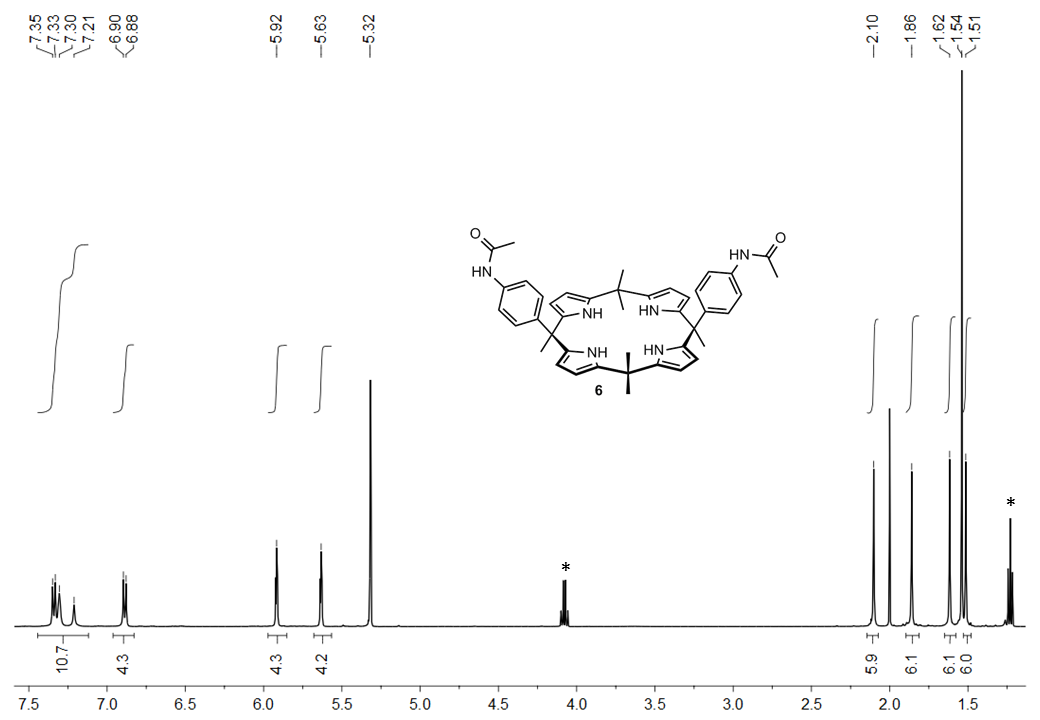


**S.I. Figure 9**. Partial ^1^H NMR (500 MHz, CD_2_Cl_2_,) spectrum of **6**. * Traces of residual EtOAc.


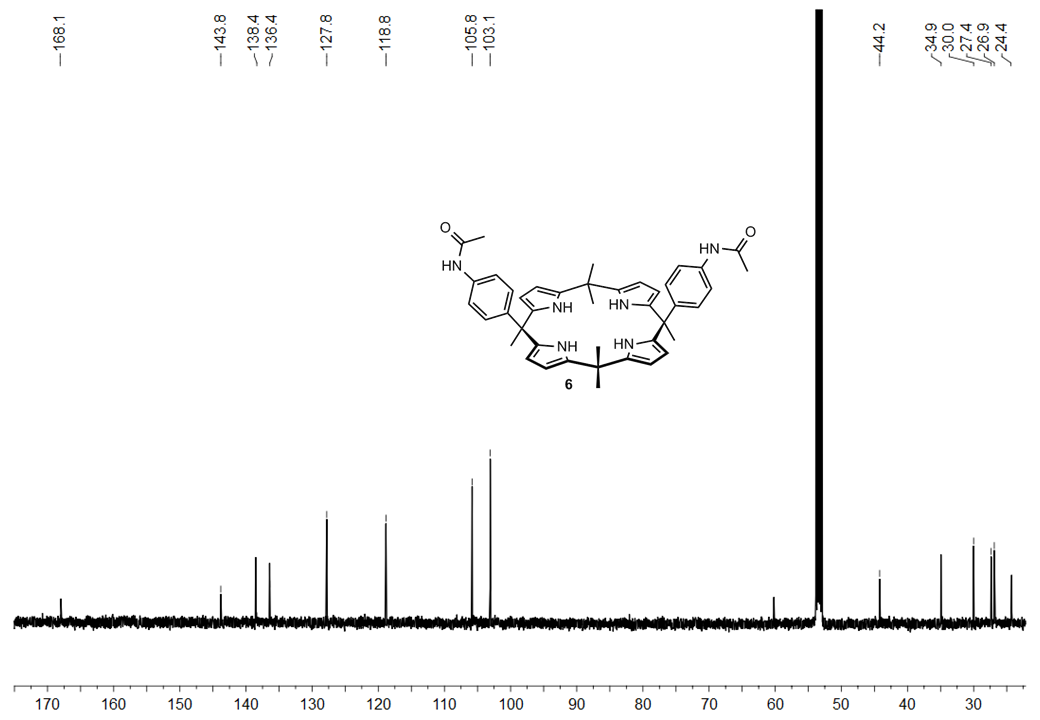


**S.I. Figure 10**. ^13^C NMR (125 MHz, CD_2_Cl_2_,) spectrum of **6**.


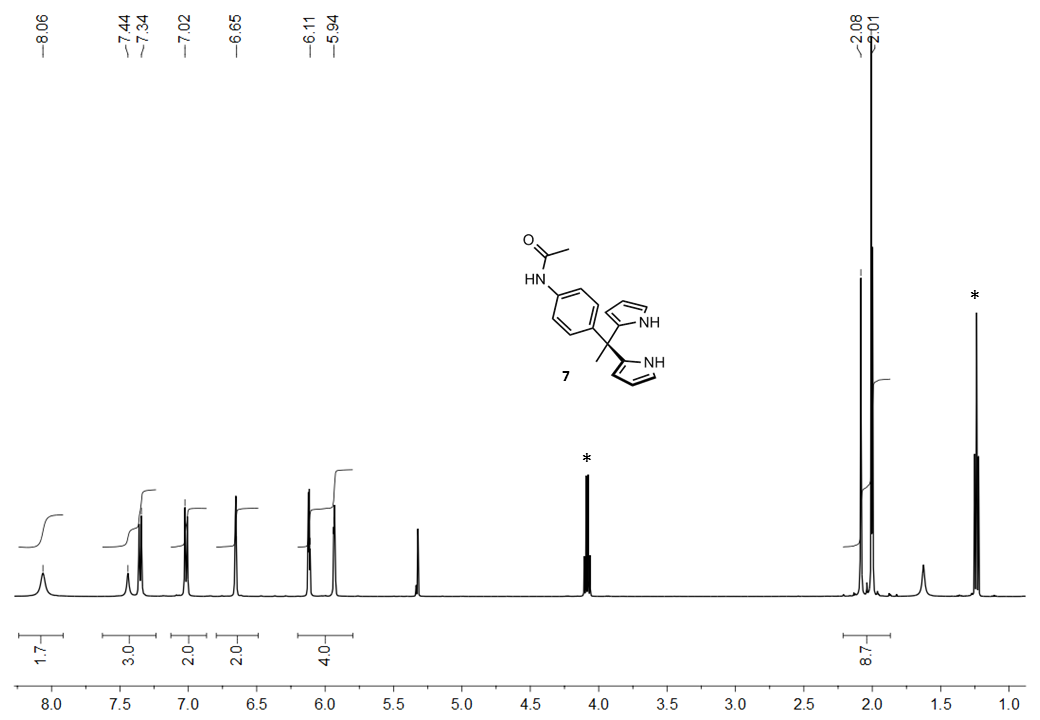


**S.I. Figure 11**. Partial ^1^H NMR (500 MHz, CD_2_Cl_2_,) spectrum of **7**. * Residual EtOAc.


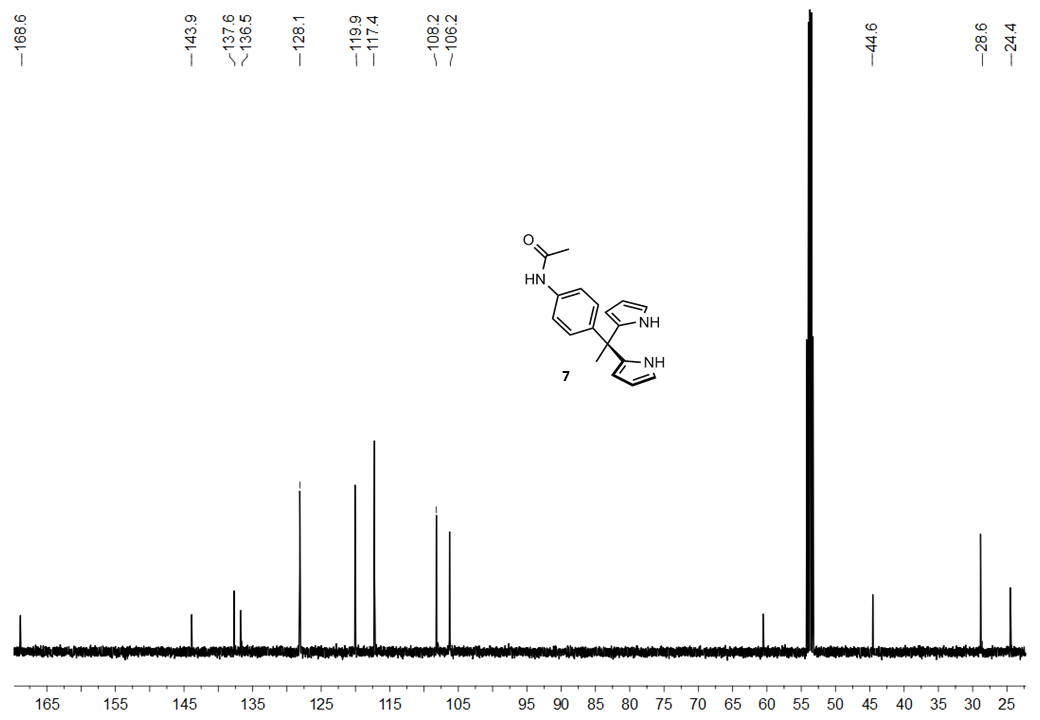


**S.I. Figure 12**. ^13^C NMR (125 MHz, CD_2_Cl_2_,) spectrum of **7**.


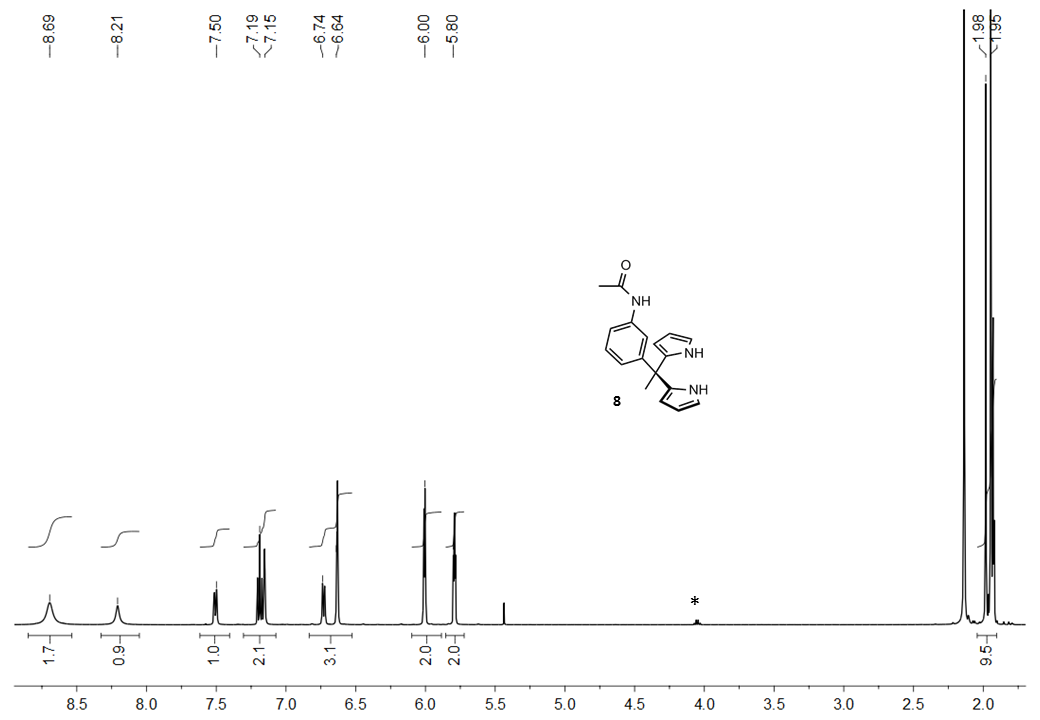


**S.I. Figure 13**. Partial ^1^H NMR (500 MHz, CD_2_Cl_2_,) spectrum of **8**. * Residual EtOAc.

**
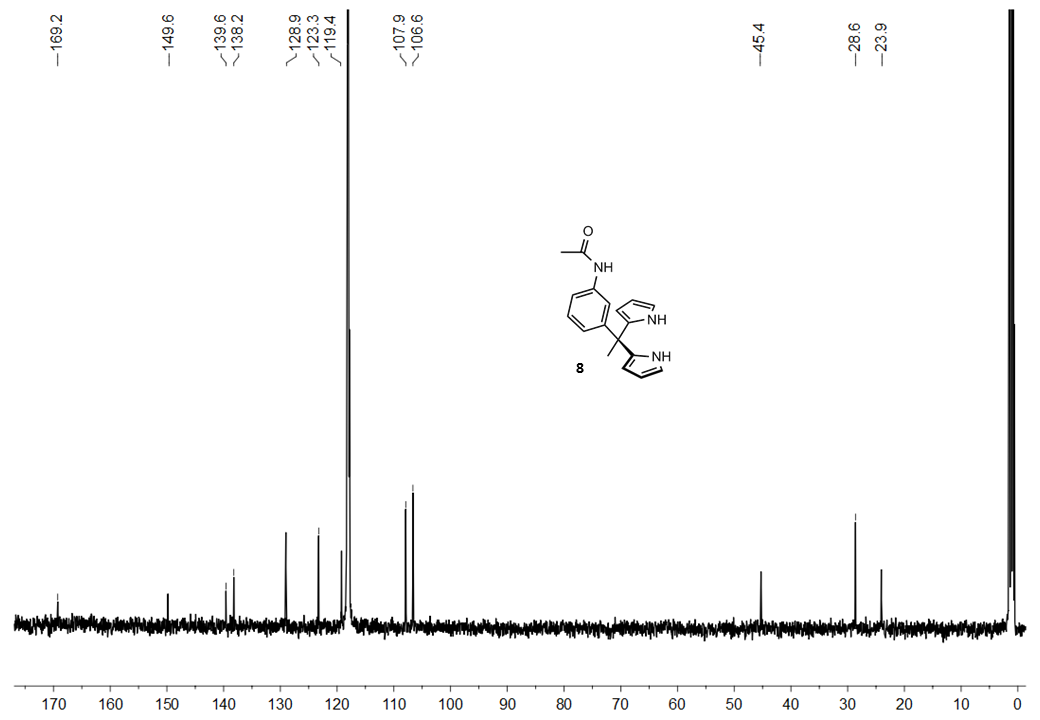
**

**S.I. Figure 14**. ^13^C NMR (125 MHz, CD_2_Cl_2_,) spectrum of **8**.

**S.I. Table 1.** microRNAs biological functions.

| **Gene Name** | **Fold change** | **Description^SI-reference^** |
| --- | --- | --- |
| hsa-miR-7-2-3p | 3.483 | c-Fos regulation ^SI^**^-^**^1^ |
| hsa-let-7b-3p/mmu-let-7b-3p/rno-let-7b-3p | 3.478 | Cell proliferation inhibition, Oncogene (RAS) suppression ^SI^**^-^**^2^ |
| hsa-let-7f-1-3p/mmu-let-7f-1-3p/rno-let-7f-1-3p | 3.117 | High Mobility Group Box 1 (HMGB1) regulation (autophagy) ^SI^**^-^**^3^ |
| hsa-miR-26a-2-3p | 2.645 | Inhibition of TGF expression ^SI^**^-^**^4^ |
| hsa-miR-26b-3p/mmu-miR-26b-3p/rno-miR-26b-3p | 2.733 | Regulation of human umbilical cord-derived mesenchymal stem cell proliferation by targeting estrogen receptor ^SI^**^-^**^5^ |
| hsa-miR-30b-5p/mmu-miR-30b-5p/rno-miR-30b-5p | 3.981 | Stress response (NF-kappaB activation), Protein repair, Cell cycle inhibition, Oncogene (EGF) activation ^SI^**^-^**^2^ |
| hsa-miR-30c-5p/mmu-miR-30c-5p/rno-miR-30c-5p | 2.873 | Targets fatty acid synthase; androgen receptor inhibitor ^SI^**^-^**^6,^ ^SI^**^-^**^7^ |
| hsa-miR-34b-3p | 3.926 | P53 effector ^SI^**^-^**^2^ |
| hsa-miR-129-1-3p/mmu-miR-129-1-3p | 4.293 | EMT transition, cell growth ^SI^**^-^**^8^ |
| hsa-miR-130b-5p/mmu-miR-130b-5p/rno-miR-130b-5p | 2.779 | Cell proliferation ^SI^**^-^**^9,^ ^SI^**^-^**^10^ |
| hsa-miR-142-5p/mmu-miR-142-5p/rno-miR-142-5p | 3.303 | Cell growth cancer inhibitor ^SI^**^-^**^11^ |
| hsa-miR-150-5p/mmu-miR-150-5p/rno-miR-150-5p | 2.966 | Proliferation rate and migration activity promotion through regulation of transcription factor Sp1 ^SI^**^-^**^12^ |
| hsa-miR-196a-3p/mmu-miR-196a-2-3p/rno-miR-196a-3p | 3.617 | Regulation of fibroblast growth factor (FGF2) ^SI^**^-^**^13^ |
| hsa-miR-197-3p | 4.166 | Apoptosis, cell proliferation ^SI^**^-^**^2^ |
| hsa-miR-203b-5p/rno-miR-203b-5p | 3.407 | Regulation of MyoD expression; tumour suppressor ^SI^**^-^**^14,^ ^SI^**^-^**^15^ |
| hsa-miR-204-5p/mmu-miR-204-5p/rno-miR-204-5p | 3.015 | Tumour suppressor;  cell proliferation ^SI^**^-^**^16,^ ^SI^**^-^**^17^ |
| hsa-miR-296-5p/mmu-miR-296-5p/rno-miR-296-5p | 0.171 | Caspase-8 (CASP8) and nerve growth factor receptor (NGFR) regulation; cell viability suppression through regulation of Polo-like kinase 1 (PLK1);  tumour suppressor targeting Peptidyl-prolyl cis-trans isomerase (pin 1) ^SI^**^-^**^18, SI^**^-^**^19, SI^**^-^**^20^ |
| hsa-miR-361-3p/mmu-miR-361-3p/rno-miR-361-3p | 3.52 | EMT transition; cell proliferation ^SI^**^-^**^21, SI^**^-^**^22^ |
| hsa-miR-372 | 0.342 | Migration and Invasion suppression by Targeting p65 in prostate cancer |
| hsa-miR-374c-5p | 3.794 | No references available |
| hsa-miR-377-3p/mmu-miR-377-3p | 2.979 | Tumour suppressor ^SI^**^-^**^23^ |
| hsa-miR-449b-3p | 2.999 | Mammalian cortex development; cell proliferation ^SI^**^-^**^24, SI^**^-^**^25^ |
| hsa-miR-545-5p | 3.325 | Cell proliferation ^SI^**^-^**^26^ |
| hsa-miR-548aa/hsa-miR-548ap-3p/hsa-miR-548t-3p | 3.500 | Antioncogenic regulator ^SI^**^-^**^27^ |
| hsa-miR-548as-3p | 2.810 | Antioncogenic regulator ^SI^**^-^**^27^ |
| hsa-miR-559 | 2.765 | ERBB2 regulation ^SI^**^-^**^28^ |
| hsa-miR-576-5p | 2.877 | ITGBL1 (integrin subunit beta like 1, member of the EGF-like protein family) regulation ^SI^**^-^**^29^ |
| hsa-miR-652-3p/mmu-miR-652-3p/rno-miR-652-3p | 0.320 | Proliferation and metastasis promotion by targeting Lgl1 (Leucine-rich, glioma inactivated 1) in lung cancer ^SI^**^-^**^30^ |
| hsa-miR-656 | 4.223 | Cell proliferation, migration and  invasion ^SI^**^-^**^31^ |
| hsa-miR-659-3p | 0.319 | Sphingosine kinase 1 (SPHK1) regulation and colorectal cancer chemo-resistance implication; focal adhesion pathway deregulation in neuroblastoma; progranulin gene (GNR) regulation ^SI^**^-^**^32, SI^**^-^**^33, SI^**^-^**^34^ |
| hsa-miR-659-5p | 3.521 | JNK2 (protein kinase of the MAPK family) regulation; GRN (progranulin, involved in the regulation of cell growth and cell cycle progression) regulation; myogenic differentiation ^SI^**^-^**^35, SI^**^-^**^36, SI^**^-^**^37^ |
| hsa-miR-660-3p | 3.257 | Lung cancer inhibitor targeting P53 pathway ^SI^**^-^**^38^ |
| hsa-miR-676-5p | 3.611 | No references available |
| hsa-miR-877-3p | 3.725 | Myofibroblast differentiation of lung resident mesenchymal stem cells;  production of IL-8 and IL-1β in mesangial cells ^SI-39, SI-40^ |

**S.I. References.**

SI-1 Zhao, X. D., Lu. Y. Y., Guo, H., Xie, H. H., He, L. J., Shen, G. F., Zhou, J. F., Li, T., Hu, S. J., Zhou, L., Han, Y. N., Liang, S. L., Wang, X., Wu, K. C., Shi, Y. Q., Nie, Y. Z. & Fan, D. M. MicroRNA-7/NF-κB signaling regulatory feedback circuit regulates gastric carcinogenesis. *J. Cell. Biol.* **210** (4), 613-627 (2015).

SI-2 Izzotti, A., Cartiglia, C., Steele, V. & De Flora, S. MicroRNAs as targets for dietary and pharmacological inhibitors of mutagenesis and carcinogenesis. *Mutat. Res. Rev.* **751**, 287-303 (2012).

SI-3 Pannuru, P., Dontula, R., Khan, A. A., Herbert, E., Ozer, H., Chetty, C. & Lakka, S. S. miR-let-7f-1 regulates SPARC mediated cisplatin resistance in medulloblastoma cells. *Cell. Signal.* **26** (10), 2193-2201 (2014).

SI-4 Koga, K., Yokoi, H., Mori, K., Kasahara, M., Kuwabara, T., Imamaki, H., Ishii, A., Mori, K. P., Kato, Y., Ohno, S., Toda, N., Saleem, M. A., Sugawara, A., Nakao, K., Yanagita, M. & Mukoyama, M. MicroRNA-26a inhibits TGF-β-induced extracellular matrix protein expression in podocytes by targeting CTGF and is downregulated in diabetic nephropathy. *Diabetologia* **58** (9), 2169-2180 (2015).

SI-5 Wang, Q., Xu, C., Zhao, Y., Xu, Z., Zhang, Y., Jiang, J., Yan, B., Gu, D., Wu, M., Wang, Y. & Liu, H. miR-26b-3p Regulates Human Umbilical Cord-Derived Mesenchymal Stem Cell Proliferation by Targeting Estrogen Receptor. *Stem Cells Dev.* **25** (5), 415-426 (2016).

SI-6 Kumar, B., Khaleghzadegan, S., Mears, B., Hatano, K., Kudrolli, T. A., Chowdhury, W. H., Yeater, D. B., Ewing, C. M., Luo, J., Isaacs, W. B., Marchionni, L. & Lupold, S. E. Identification of miR-30b-3p and miR-30d-5p as direct regulators of androgen receptor signaling in prostate cancer by complementary functional microRNA library screening. *Oncotarget* **7** (45), 72593-72607 (2016).

SI-7 Fan, J., Li, H., Nie, X., Yin, Z., Zhao, Y., Chen, C. & Wang, D. W. MiR-30c-5p ameliorates hepatic steatosis in leptin receptor-deficient (db/db) mice via down-regulating FASN. *Oncotarget* **8** (8), 13450-13463 (2017).

SI-8 Liu, M. X., Zhou, K. C. & Cao, Y. MCRS1 overexpression, which is specifically inhibited by miR-129*, promotes the epithelial-mesenchymal transition and metastasis in non-small cell lung cancer. *Mol. Cancer* **13** (1), 245 (2014).

SI-9 Gu, J. J., Zhang, J. H., Chen, H. J. & Wang, S.S. MicroRNA-130b promotes cell proliferation and invasion by inhibiting peroxisome proliferator-activated receptor-γ in human glioma cells. *Int. J. Mol. Med.* **37** (6), 1587-1593 (2016).

SI-10 Chang, R. M., Xu, J. F., Fang, F., Yang, H. & Yang, L. Y. MicroRNA-130b promotes proliferation and EMT-induced metastasis via PTEN/p-AKT/HIF-1α signaling. *Tumor Biol.* **37** (8), 10609-10619 (2016); doi:10.1007/s13277-016-4919-z..

SI-11 Lou, K., Chen, N., Li, Z., Zhang, B., Wang, X., Chen, Y., Xu, H., Wang, D. & Wang, H. MicroRNA-142-5p Overexpression Inhibits Cell Growth and Induces Apoptosis by Regulating FOXO in Hepatocellular Carcinoma Cells. *Oncol. Res*. **25** (1), 65-73 (2017).

SI-12 Xia, B., Hou, Y., Chen, H., Yang, S., Liu, T., Lin, M. & Lou, G. Long non-coding RNA ZFAS1 interacts with miR-150-5p to regulate Sp1 expression and ovarian cancer cell malignancy. *Oncotarget* **8** (12), 19534-19546 (2017); doi: 10.18632/oncotarget.14663.

SI-13 Zhu, D. L., Guo, Y., Zhang, Y., Dong, S. S., Xu, W., Hao, R. H., Chen, X. F., Yan, H., Yang, S. Y. & Yang, T. L. A functional SNP regulated by miR-196a-3p in the 3'UTR of FGF2 is associated with bone mineral density in the Chinese Population. *Hum. Mutat.* **38** (6), 725-735 (2017); doi:10.1002/humu.23216.

SI-14 Yan, B., Guo, J. T., Zhu, C. D., Zhao, L. H. & Zhao, J. L. miR-203b: a novel regulator of MyoD expression in tilapia skeletal muscle. *J. Exp. Biol.* **216** (3), 447-451 (2013).

SI-15 Liu, W., Dong, Z., Liang, J., Guo, X., Guo, Y., Shen, S., Kuang, G. & Guo, W. Downregulation of Potential Tumor Suppressor miR-203a by Promoter Methylation Contributes to the Invasiveness of Gastric Cardia Adenocarcinoma. *Cancer Invest.* **34** (10), 506-516 (2016).

SI-16 Wang, X., Li, F. & Zhou, X. miR-204-5p regulates cell proliferation and metastasis through inhibiting CXCR4 expression in OSCC. *Biomed. Pharmacother.* **82** (8), 202-207 (2016).

SI-17 Luan, W., Qian, Y., Ni, X., Bu, X., Xia, Y., Wang, J., Ruan, H., Ma, S. & Xu, B. miR-204-5p acts as a tumor suppressor by targeting matrix metalloproteinases-9 and B-cell lymphoma-2 in malignant melanoma. *Onco Targets Ther.* **10** (2), 1237-1246 (2017).

SI-18 Lee, K. H., Lin, F. C., Hsu, T. I., Lin, J. T., Guo, J. H., Tsai, C. H., Lee, Y. C., Lee, Y. C., Chen, C. L., Hsiao, M. & Lu, P. J. MicroRNA-296-5p (miR-296-5p) functions as a tumor suppressor in prostate cancer by directly targeting Pin1. *Biochim. Biophys. Acta, Mol. Cell. Res.* **1843** (9), 2055-2066 (2014).

SI-19 Lee, H., Shin, C. H., Kim, H. R., Choi, K. H. & Kim, H. H. MicroRNA-296-5p Promotes Invasiveness through Downregulation of Nerve Growth Factor Receptor and Caspase-8. *Mol Cells.***40** (4), 254 (2017); doi: 10.14348/molcells.2017.2270.

SI-20 Xu, C., Li, S., Chen, T., Hu, H., Ding, C., Xu, Z., Chen, J., Liu, Z., Lei, Z., Zhang, H. T., Li, C. & Zhao, J. miR-296-5p suppresses cell viability by directly targeting PLK1 in non-small cell lung cancer. *Oncol. Rep.* **35** (1), 497-503 (2016).

SI-21 Chen, W., Wang, J., Liu, S., Wang, S., Cheng, Y., Zhou, W., Duan, C. & Zhang, C. MicroRNA-361-3p suppresses tumor cell proliferation and metastasis by directly targeting SH2B1 in NSCLC. *J. Exp. Clin. Cancer Res.* **35** (1), 76 (2016).

SI-22 Zhang, X., Wei, C., Li, J., Liu, J. & Qu, J. MicroRNA-361-5p inhibits epithelial-to-mesenchymal transition of glioma cells through targeting Twist1. *Oncology Reports* **37** (3), 1849-1856 (2017); doi: 10.3892/or.2017.5406.

SI-23 Sun, C., Li, S., Zhang, F., Xi, Y., Wang, L., Bi, Y. & Li, D. Long non-coding RNA NEAT1 promotes non-small cell lung cancer progression through regulation of miR-377-3p-E2F3 pathway. *Oncotarget* **7** (32), 51784-51844 (2016).

SI-24 Bou, Kheir, T., Futoma-Kazmierczak, E., Jacobsen, A., Krogh, A., Bardram, L., Hother, C., Grønbæk, K., Federspiel, B., Lund, A. H. & Friis-Hansen, L. miR-449 inhibits cell proliferation and is down-regulated in gastric cancer. *Mol Cancer.* **10** (1), 29 (2011).

SI-25 Fededa, J. P., Esk, C., Mierzwa, B., Stanyte, R., Yuan, S., Zheng, H., Ebnet, K., Yan, W., Knoblich, J. A. & Gerlich, D. W. MicroRNA-34/449 controls mitotic spindle orientation during mammalian cortex development. *EMBO J.* **35** (22), 2386-2398 (2016).

SI-26 Huang X, Lu S. MicroR-545 mediates colorectal cancer cells proliferation through up-regulating epidermal growth factor receptor expression in HOTAIR long non-coding RNA dependent. *Mol. Cell. Biochem.* **431** (1-2), 1-10 (2017); doi: 10.1007/s11010-017-2974-4.

SI-27 Shi, Y., Qiu, M., Wu, Y. & Hai, L. MiR-548-3p functions as an anti-oncogenic regulator in breast cancer. *Biomed. Pharmacother.* **75**, 111-116 (2015).

SI-28 Chen, H., Sun, J. G., Cao, X.W., Ma, X. G., Xu, J. P., Luo, F. K. & Chen, Z. T. Preliminary validation of ERBB2 expression regulated by miR-548d-3p and miR-559. *Biochem. Biophys. Res. Commun.* **385** (4), 596-600 (2009).

SI-29 Gan, X., Liu, Z., Tong, B. & Zhou, J. Epigenetic downregulated ITGBL1 promotes non-small cell lung cancer cell invasion through Wnt/PCP signaling. *Tumour Biol.* **37** (2), 1663-1669 (2016).

SI-30 Yang, W., Zhou, C., Luo, M., Shi, X., Li, Y., Sun, Z., Zhou, F., Chen, Z. & He, J. MiR-652-3p is upregulated in non-small cell lung cancer and promotes proliferation and metastasis by directly targeting Lgl1. *Oncotarget* **7** (13), 16703-16715 (2016).

SI-31 Guo, M., Jiang, Z., Zhang, X., Lu, D.,, Ha, A. D., Sun, J., Du, W., Wu, Z., Hu, L., Khadarian, K., Shen, J. & Lin, Z. miR-656 inhibits glioma tumorigenesis through repression of BMPR1A. *Carcinogenesis* **35** (8), 1698-1706 (2014).

SI-32 Li, S., Fang, Y., Qin, H., Fu, W. & Zhang, X. miR-659-3p is involved in the regulation of the chemotherapy response of colorectal cancer via modulating the expression of SPHK1. *Am. J. Cancer Res*. **6** (9), 1976-1985 (2016).

SI-33 Stigliani, S., Scaruffi, P., Lagazio, C., Persico, L., Carlini, B., Varesio, L., Morandi, F., Morini, M., Gigliotti, A. R., Esposito, M. R., Viscardi, E., Cecinati, V., Conte, M. & Corrias, M. V. Deregulation of focal adhesion pathway mediated by miR-659-3p is implicated in bone marrow infiltration of stage M neuroblastoma patients. *Oncotarget* **6** (15) 13295-13308 (2015).

SI-34 Piscopo, P., Grasso, M., Fontana, F., Crestini, A., Puopolo, M., Del Vescovo, V., Venerosi, A., Calamandrei, G., Vencken, S. F., Greene, C. M., Confaloni, A. & Denti, M. A. Reduced miR-659-3p Levels Correlate with Progranulin Increase in Hypoxic Conditions: Implications for Frontotemporal Dementia. *Front. Mol. Neurosci.* **9**, 31 (2016); doi: 10.3389/fnmol.2016.00031

SI-35 Rademakers, R., Eriksen, J. L., Baker, M., Robinson, T., Ahmed, Z., Lincoln, S. J., Finch, N., Rutherford, N. J., Crook, R. J., Josephs, K. A., Boeve, B. F., Knopman, D. S., Petersen, R. C., Parisi, J. E., Caselli, R. J., Wszolek, Z. K., Uitti, R. J., Feldman, H., Hutton, M. L., Mackenzie, I. R., Graff-Radford, N. R. & Dickson, D. W. Common variation in the miR-659 binding-site of GRN is a major risk factor for TDP43-positive frontotemporal dementia. *Hum. Mol. Genet.* **17** (23), 3631-3642 (2008).

SI-36 Dmitriev, P., Barat, A., Polesskaya, A., O'Connell, M. J., Robert, T., Dessen, P., Walsh, T. A., Lazar, V., Turki, A., Carnac, G., Laoudj-Chenivesse, D., Lipinski, M. & Vassetzky, Y. S. Simultaneous miRNA and mRNA transcriptome profiling of human myoblasts reveals a novel set of myogenic differentiation-associated miRNAs and their target genes. *BMC Genomics* **14** (1), 265 (2013).

SI-37 Luo, G., Zhou, Y., Yi, W. & Yi, H. Expression levels of JNK associated with polymorphic lactotransferrin haplotypes in human nasopharyngeal carcinoma. *Oncol. Lett.* **12** (2), 1085-1094 (2016).

SI-38 Borzi, C., Calzolari, L., Centonze, G., Milione, M., Sozzi, G. & Fortunato, O. mir-660-p53-mir-486 Network: A New Key Regulatory Pathway in Lung Tumorigenesis. *Int. J. Mol. Sci.* **18** (1), 222 (2017).

SI-39 Wang, C., Gu, S., Cao, H., Li, Z,, Xiang, Z., Hu, K. & Han, X. miR-877-3p targets Smad7 and is associated with myofibroblast differentiation and bleomycin-induced lung fibrosis. *Sci. Rep.* **6**, 30122 (2016).

SI-40 Liang, Y., Zhao, G., Tang, L., Zhang, J., Li, T. & Liu, Z. MiR-100-3p and miR-877-3p regulate overproduction of IL-8 and IL-1β in mesangial cells activated by secretory IgA from IgA nephropathy patients. *Exp. Cell. Res.* **347** (2), 312-321 (2016).
